# *GLIS3*, a Susceptibility Gene for Type 1 and Type 2 Diabetes, Modulates Pancreatic Beta Cell Apoptosis via Regulation of a Splice Variant of the BH3-Only Protein *Bim*

**DOI:** 10.1371/journal.pgen.1003532

**Published:** 2013-05-30

**Authors:** Tatiane C. Nogueira, Flavia M. Paula, Olatz Villate, Maikel L. Colli, Rodrigo F. Moura, Daniel A. Cunha, Lorella Marselli, Piero Marchetti, Miriam Cnop, Cécile Julier, Decio L. Eizirik

**Affiliations:** 1 Laboratory of Experimental Medicine, Medical Faculty, Université Libre de Bruxelles, Brussels, Belgium; 2 Department of Endocrinology and Metabolism, University of Pisa, Pisa, Italy; 3 Division of Endocrinology, Erasmus Hospital, Brussels, Belgium; 4 INSERM UMR-S 958, Faculté de Médecine Paris Diderot, Paris, France; 5 University Paris 7 Denis–Diderot, Paris, France; University of Oxford, United Kingdom

## Abstract

Mutations in human Gli-similar (GLIS) 3 protein cause neonatal diabetes. The GLIS3 gene region has also been identified as a susceptibility risk locus for both type 1 and type 2 diabetes. GLIS3 plays a role in the generation of pancreatic beta cells and in insulin gene expression, but there is no information on the role of this gene on beta cell viability and/or susceptibility to immune- and metabolic-induced stress. GLIS3 knockdown (KD) in INS-1E cells, primary FACS-purified rat beta cells, and human islet cells decreased expression of MafA, Ins2, and Glut2 and inhibited glucose oxidation and insulin secretion, confirming the role of this transcription factor for the beta cell differentiated phenotype. GLIS3 KD increased beta cell apoptosis basally and sensitized the cells to death induced by pro-inflammatory cytokines (interleukin 1β + interferon-γ) or palmitate, agents that may contribute to beta cell loss in respectively type 1 and 2 diabetes. The increased cell death was due to activation of the intrinsic (mitochondrial) pathway of apoptosis, as indicated by cytochrome *c* release to the cytosol, Bax translocation to the mitochondria and activation of caspases 9 and 3. Analysis of the pathways implicated in beta cell apoptosis following GLIS3 KD indicated modulation of alternative splicing of the pro-apoptotic BH3-only protein Bim, favouring expression of the pro-death variant Bim_S_ via inhibition of the splicing factor SRp55. KD of Bim abrogated the pro-apoptotic effect of GLIS3 loss of function alone or in combination with cytokines or palmitate. The present data suggest that altered expression of the candidate gene *GLIS3* may contribute to both type 1 and 2 type diabetes by favouring beta cell apoptosis. This is mediated by alternative splicing of the pro-apoptotic protein Bim and exacerbated formation of the most pro-apoptotic variant Bim_S_.

## Author Summary

Pancreatic beta cell dysfunction and death is a central event in the pathogenesis of diabetes. Genome-wide association studies have identified a large number of associations between specific loci and the two main forms of diabetes, namely type 1 and type 2 diabetes, but the mechanisms by which these candidate genes predispose to diabetes remain to be clarified. The *GLIS3* gene region has been identified as a susceptibility risk locus for both type 1 and type 2 diabetes—it is actually the only locus showing association with both forms of diabetes and the regulation of blood glucose. We show that decreased expression of *GLIS3* may contribute to diabetes by favouring beta cell apoptosis. This is mediated by the mitochondrial pathway of apoptosis, activated via alternative splicing (a process by which exons are joined in multiple ways, leading to the generation of several proteins by a single gene) of the pro-apoptotic protein Bim, which favours formation of the most pro-apoptotic variant. The present data provides the first evidence that a susceptibility gene for diabetes may contribute to disease via regulation of alternative splicing of a pro-apoptotic gene in pancreatic beta cells.

## Introduction

The Kruppel-like zinc finger protein Gli-similar (GLIS) 3 plays a critical role in pancreatic development, and loss-of-function mutations in this transcription factor lead to a syndrome characterized by neonatal diabetes, hypothyroidism and other congenital dysfunctions [1], [2]. Genome-wide association studies in large numbers of individuals with type 1 (T1D) or type 2 (T2D) diabetes indicated that common variants near *GLIS3* gene are associated with both forms of diabetes [3]–[7], making *GLIS3* one of the few candidate genes for both T1D and T2D. It remains to be proven, however, that susceptibility alleles for T1D and T2D actually decrease expression of GLIS3 in pancreatic beta cells. GLIS3 is also implicated in the regulation of human fasting glucose and insulin [4], [8] and glucose-stimulated insulin release [5], suggesting a key role for the transcription factor in human beta cell development/function.

*GLIS3* deficient mice have a major decrease in beta cell mass and develop neonatal diabetes [9], [10]. These mice also have decreased expression of several key transcription factors required for the endocrine development of the pancreas, *i.e.* Neurogenin3, NeuroD1, MafA and Pdx1 [9], [10]. Moreover, conditional knockout of GLIS3 in adult mice causes defective insulin secretion and increase susceptibility to high fat diet-induced diabetes [11]. *In vitro* knockdown (KD) or overexpression of *GLIS3* in rat insulinoma 832/13 cells showed that the transcription factor binds to a *cis*-acting element in the rat insulin 2 (*Ins2*), modulating its transcriptional activity [12]. GLIS3 also synergizes with the beta cell transcription factors Pdx1, MafA and NeuroD1, increasing insulin promoter activity, besides directly regulating the expression of *MafA* (another important inducer of the insulin promoter) [12]. These observations suggest that GLIS3 plays an important role for the development of mature pancreatic beta cells and for the transcription of its key hormone insulin.

There is, however, little information on the role of GLIS3 in beta cell susceptibility to immune- or metabolic-induced apoptosis and little data on its impact on adult beta cells. Beta cell apoptosis contributes to the two main forms of diabetes [13], [14]. Diabetes candidate genes expressed in beta cells may have a major impact on cell survival/function in T2D [15], [16], [17] and T1D [18]–[22] and in the local inflammatory responses leading to insulitis and chronic autoimmunity in T1D [19], [21], [23].

We have presently developed an *in vitro* model of *GLIS3* deficiency in beta cells by using siRNAs targeting different regions of the *GLIS3* mRNA. *GLIS3* KD increased beta cell apoptosis under basal condition and sensitized cells to death induced by interleukin 1β (IL-1β) + interferon-γ (IFN-γ) or palmitate, agents that may contribute to beta cell loss in respectively T1D and T2D. This increase in apoptosis was secondary to the activation of the intrinsic pathway of apoptosis through alternative splicing of the pro-apoptotic BH3-only protein Bim at least in part via inhibition of the splicing factor SRp55. The present data provide the first indication that a candidate gene for diabetes may modify alternative splicing and thus hamper beta cell survival.

## Results

**Figure 1:**
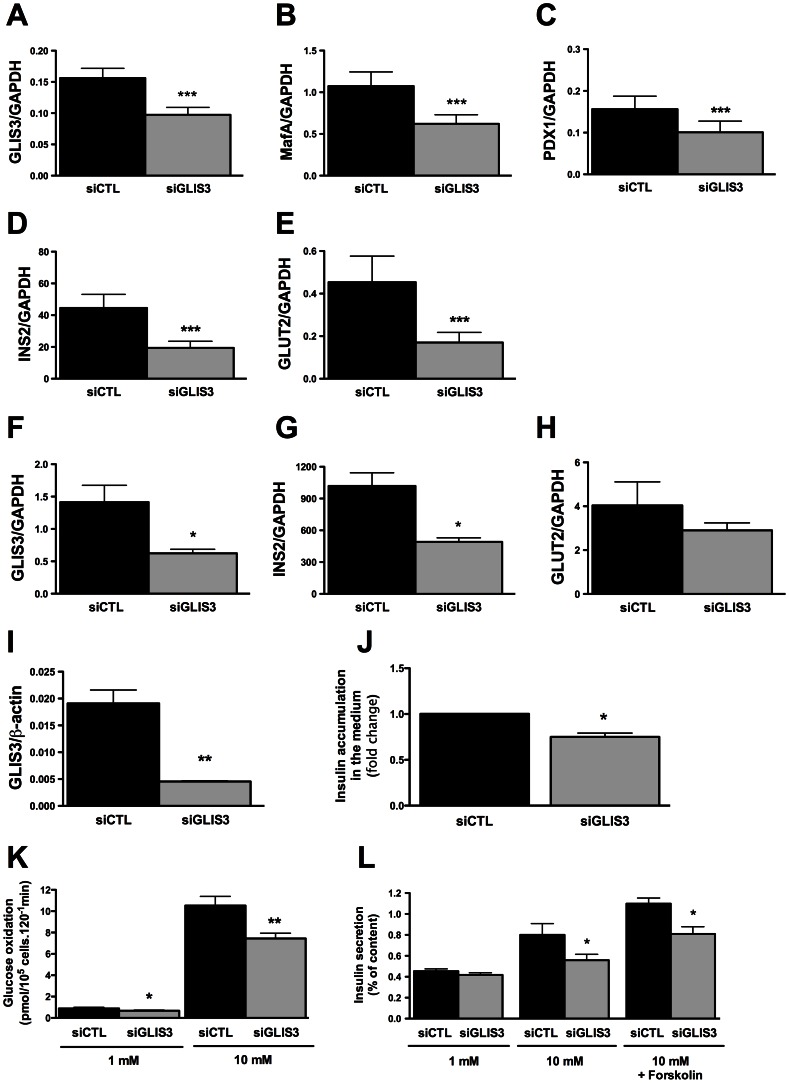
*GLIS3* regulates the differentiated beta cell phenotype. INS-1E cells, primary FACS-purified rat beta cells and human islet cells were transfected with control or GLIS3 siRNA (siCTL and siGLIS3, respectively; siGLIS3 here and below refers always to the GLIS3 siRNA#1; different siRNAs were used for rat and human cells as shown in Table S1). After 48 h, cells were used for real-time PCR analyses in INS-1E (A–E), primary rat beta cells (F–H), human islet cells (I) or functional studies (J–L). Results are means ± SEM corrected by the housekeeping genes *GAPDH* or β-actin (n = 4–5); (J) medium insulin accumulation of dispersed human islet cells; (K) glucose metabolism of INS-1E cells exposed to 1 or 10 mM glucose after *GLIS3* KD (n = 6); (L) insulin secretion in INS-1E cells treated with 1 mM glucose, 10 mM glucose or 10 mM glucose plus forskolin (20 µM) after *GLIS3* KD (n = 5). * *P*<0.05, ** *P*<0.01 and *** *P*<0.001 *vs*. siCTL by paired *t*-test.

*GLIS3* KD in INS-1E cells (Figure 1A–1E) significantly decreased key transcription factors for the maintenance of the beta cell phenotype, namely MafA and Pdx1, the glucose transporter Glut2 and INS2. These observations were reproduced using a second siRNA targeting *GLIS3* (Figure S1A and S1B), and were confirmed in primary rat beta cells, where a 50% KD of *GLIS3* led to a decrease in *INS2* expression and a trend for decreased *Glut2* expression (Figure 1F–1H). These changes in gene expression by *GLIS3* KD had a functional impact, with decreased basal and glucose-stimulated glucose metabolism and of glucose +/− forskolin-induced insulin release in INS-1E cells (Figure 1K–1L) and a 25% decrease in insulin accumulation in the medium of human islets transfected with GLIS3 siRNA, as compared to controls (Figure 1J).

**Figure 2:**
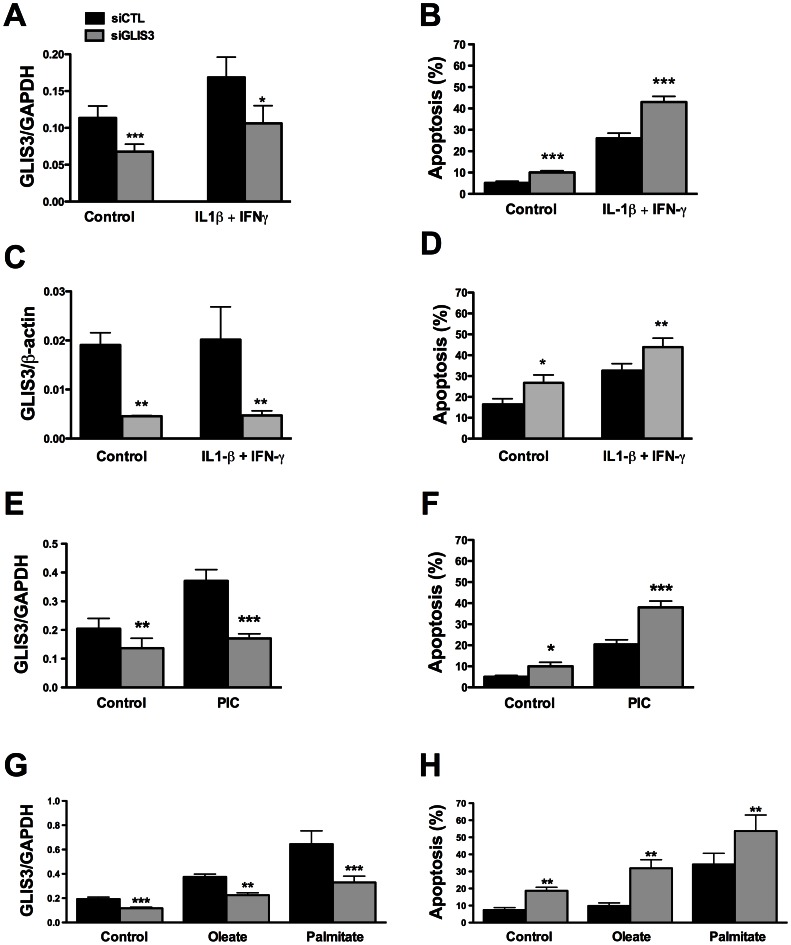
*GLIS3* KD potentiates apoptosis induced by cytokines, PIC, and FFAs. Following transfection with siCTL and siGLIS3 as in Figure 1, INS-1E cells (A, B, E–H) and human islet cells (C, D) were exposed to cytokines (A, B, C, D) (n = 4–7), PIC (E, F) (n = 7), oleate or palmitate (G, H) (n = 5). After 24 h *GLIS3* mRNA expression and apoptosis were evaluated. Results are means ± SEM. * *P*<0.05, ** *P*<0.01 or *** *P*<0.001 *vs*. siCTL by paired *t*-test.

**Figure 3:**
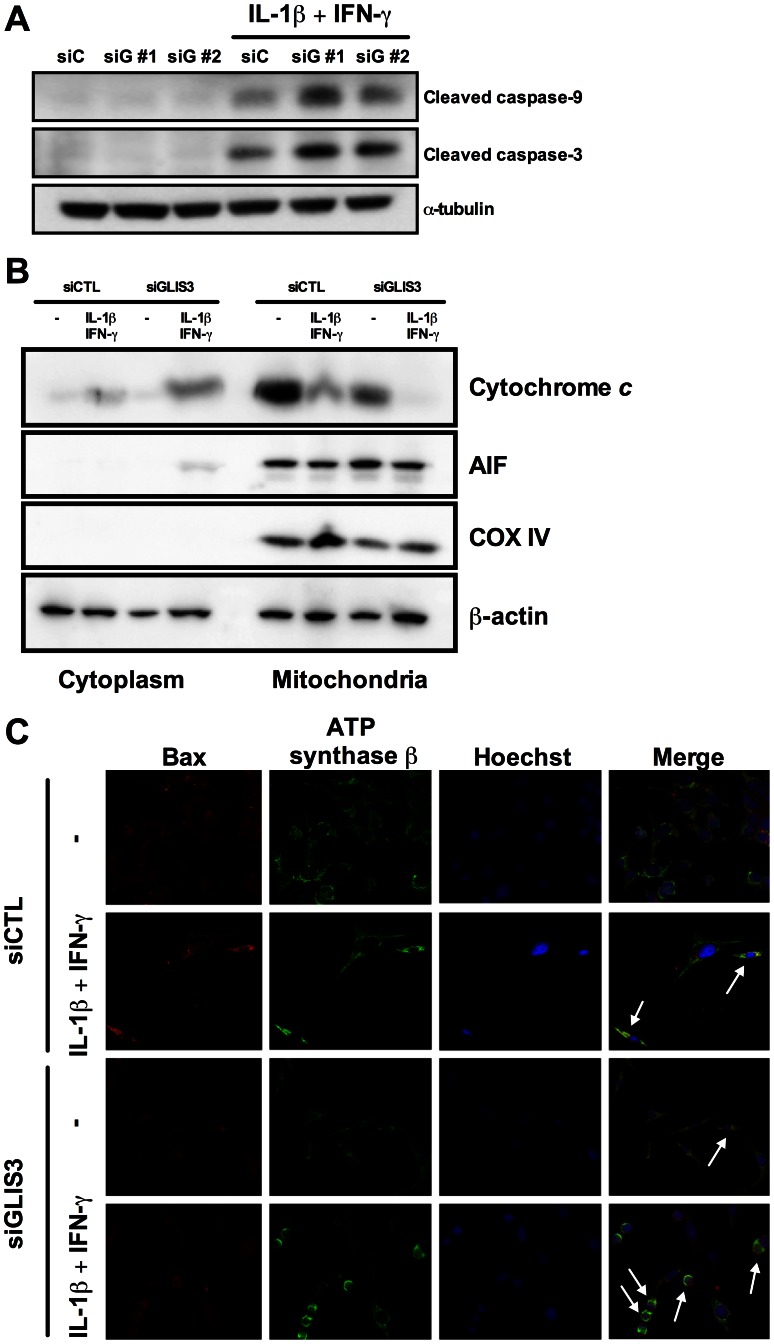
*GLIS3* KD potentiates cytokine-induced beta cell death via the mitochondrial pathway of apoptosis. INS-1E cells were transfected with siCTL or siGLIS3 (siGLIS3#1 and #2) and then exposed or not to cytokines. After 24 h cells were used for immunoblotting or immunofluorescence analysis. (A) Cleaved caspases-9 and -3. Blots are representative of 4 independent experiments. α-Tubulin was used as a control for protein loading in the different lanes; (B) cytochrome *c* release from the mitochondria to the cytosol. Blots are representative of 4 independent experiments. AIF and COX IV are used as mitochondrial markers, confirming adequate sub-cellular fractionation; (C) BAX localization was studied by immunocytochemistry. Nuclear morphology is shown by Hoechst staining. Arrows indicate BAX co-localization with ATP synthase β (mitochondrial marker). Images are representative of 4 independent experiments.

We next evaluated whether *GLIS3* KD affects beta cell viability under basal condition or following exposure to stress signals that may be relevant for type 1 diabetes, namely the pro-inflammatory cytokines IL-1β + IFN-γ or the viral by-product double stranded RNA (dsRNA) [18], [19], [21], tested here as the synthetic analog PIC, or for type 2 diabetes, namely the free fatty acids oleate and palmitate [13]. *GLIS3* KD by two independent siRNAs increased basal and cytokine-induced apoptosis in INS-1E cells (Figure 2B, Figure S1C, Figure S2A and S2B). Importantly, *GLIS3* KD by two independent siRNAs also augmented apoptosis in human islet cells, under both basal condition and following exposure to IL-1β + IFN-γ (Figure 2C and 2D, Figure S1D and S1E). The KD of *GLIS3* (Figure 2E and 2G) also sensitized INS-1E cells to apoptosis induced by PIC (Figure 2F), oleate and palmitate (Figure 2H). Thus, even a partial decrease in *GLIS3* expression, as may be the case in some of the diabetes-predisposing gene polymorphisms, enhances beta cell sensitivity to basal, immune- or metabolic stress-induced apoptosis. In a mirror image of these experiments, *GLIS3* overexpression using an adenoviral vector (Figure S3A) lead to increase MafA expression (Figure S3B) and decreased by >50% cytokine-induced apoptosis in INS-1E cells (Figure S3C). Apoptosis secondary to *GLIS3* KD and exposure to pro-inflammatory cytokines was mediated by the intrinsic (mitochondrial) pathway of apoptosis, as suggested by increased cleavage of caspases 9 and 3 (Figure 3A; densitometry in Figure S2A and S2B), cytochrome *c* release to the cytosol (Figure 3B; densitometry in Figure S2C) and Bax translocation to the mitochondria (Figure 3C).

A possible mechanism for cytokine-induced apoptosis in beta cells is increased nitric oxide production and consequent endoplasmic reticulum (ER) stress and Chop activation [24], [25]. *GLIS3* KD, however, did not increase nitric oxide production (Figure S4A) or *Chop* expression (Figure S4B), making it unlikely that these are relevant mechanisms for beta cell apoptosis following *GLIS3* inhibition. Interestingly, *GLIS3* KD led to a decrease in *Chop* expression under basal condition or at some time points following cytokine exposure. Beta cells express markers of ER stress even under basal condition, probably due to the high load on the ER caused by physiological and fluctuating insulin production [26]. It is conceivable that the decrease in *Ins2* mRNA expression observed in *GLIS3* KD cells (Figure 1D) contributes to the observed decrease in *Chop* expression.

**Figure 4:**
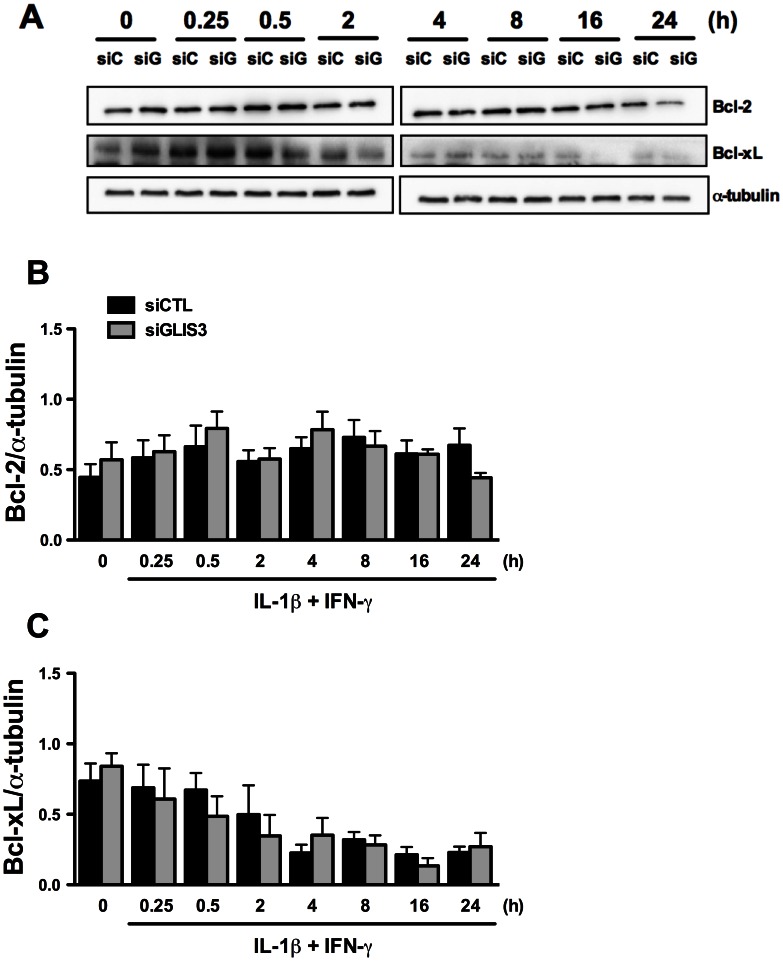
*GLIS3* KD does not change expression of the anti-apoptotic proteins Bcl-2 and Bcl-xL. INS-1E cells were transfected with control or GLIS3 siRNA. After 48 h cells were incubated with cytokines and collected at different time points for Western blot analyses. Representative blots (A) and densitometry (B, C) of Bcl-2 and Bcl-xL protein expression normalized by the housekeeping protein α-tubulin. Results are means ± SEM (n = 4).

Beta cell survival is critically dependent on the balance between anti- and pro-apoptotic Bcl-2 proteins [27]. To examine whether *GLIS3* modulates these proteins we measured expression of two key anti-apoptotic proteins, namely Bcl-2 and Bcl-xL. *GLIS3* inhibition did not affect Bcl-2 and Bcl-xL expression under basal condition or following exposure to cytokines (Figure 4), and neither was there a change in a third anti-apoptotic protein, namely Mcl-1 (data not shown). We next examined the pro-apoptotic BH3-only proteins DP5 and PUMA. These proteins have previously been shown to contribute to IL-1β + IFN-γ-mediated beta cell apoptosis [28], [29], but their expression was not increased by *GLIS3* KD (Figure S4C and S4D). If anything, there was a decrease in *PUMA* expression at some time points.

**Figure 5:**
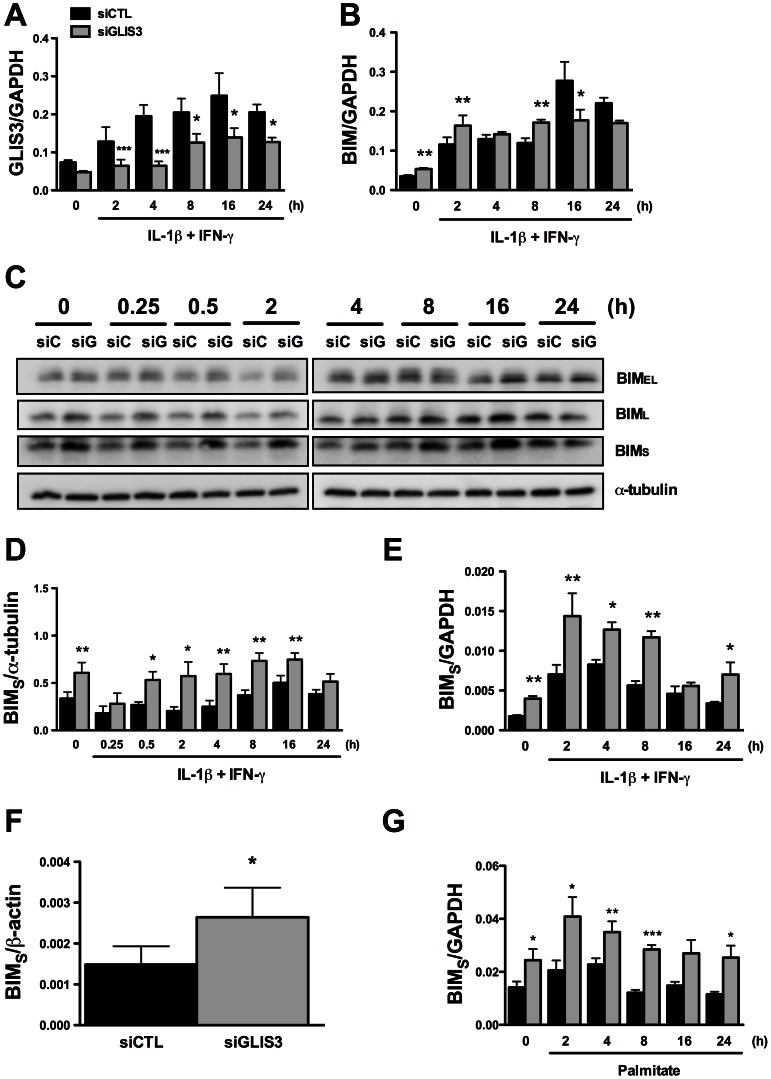
*GLIS3* KD induces *Bim* expression in INS-1E and human dispersed islet cells. INS-1E (A–E, G) and human dispersed islet cells (F) were transfected with control or GLIS3 siRNA. After 48 h human islets were collected for real-time analysis and INS-1E cells were incubated with cytokines (A–E) or palmitate (G) and collected at different time points for Western blot or real-time PCR analyses. mRNA expression of *GLIS3* (A) and *Bim* (B) after *GLIS3* KD; (C) representative blot (n = 4) of the expression of the protein isoforms Bim_EL_ (extra-large), Bim_L_ (large) and Bim_S_ (small); (D) densitometry of Bim_S_ expression normalized by the housekeeping protein α-tubulin; (E, F and G) mRNA expression of *Bim_S_* after *GLIS3* KD in INS-1E cells and exposure to cytokines (E) or palmitate (G) or in human dispersed islet cells under basal conditions (F). Results are means ± SEM (n = 4). * *P*<0.05, ** *P*<0.01 and *** *P*<0.001 *vs*. siCTL. Paired *t*-test.

Another important mediator of cytokine-induced beta cell apoptosis is the BH3-only protein Bim. Previous studies from our group have shown that STAT-1-induced Bim expression [30], [31] and JNK-induced Bim phosphorylation on serine 65 [20] contribute to beta cell apoptosis. *GLIS3* KD (Figure 5A) increased basal *Bim* mRNA expression and led to a mild increase in its expression following cytokine treatment at 2 and 8 h, with a decrease after 16 and 24 h (Figure 5B). This was independent of STAT-1 activation, since *GLIS3* KD did not modify total or phospho-STAT1 expression following exposure to IL-1β + IFN-γ for 0.25–24 h (data not shown). *Bim* has three main isoforms generated by alternative splicing, namely *Bim_EL_*, *Bim_L_*, and *Bim_S_* [32]. Western blot showed a preferential and nearly 2-fold increase in the expression of Bim_S_ in *GLIS3* KD cells both before and after exposure to cytokines (Figure 5C; the blots are quantified in Figure 5D). There was a less marked increase in Bim_EL_ and Bim_L_ at some of the time points following cytokine exposure (Figure 5C; densitometry in Figure S5A and S5B). The *Bim_S_* up-regulation seems to be secondary to GLIS3-modulated alternative splicing, since *GLIS3* KD induced a nearly 2-fold increase in *Bim_S_* mRNA expression basally and following cytokine exposure (Figure 5E), with only a minor and transient increase in *Bim_EL_* and *Bim_L_* mRNA (at 2–8 h of cytokine treatment) which was followed by a significant decrease after 16 and 24 h (Figure S5C and S5D). Importantly, these findings were reproduced in human islets, where KD of GLIS3 led to nearly 50% increase of *Bim_S_* (Figure 5F) with no significant increase in the other two splice variants (Figure S5E and S5F). In INS-1E cells exposed to palmitate, there was also a significant increase in *Bim_S_* expression (Figure 5G) and less marked changes in *Bim_EL_* and *Bim_L_* (Figure S5G and S5H). The mirror image was seen in gain-of-function experiments: adenoviral *GLIS3* overexpression (Figure S7A) decreased Bim_S_ expression and caspase 3 cleavage (Figure S7B). The decreased caspase 3 activation corroborates the finding that *GLIS3* overexpression protects against cytokine-induced apoptosis (Figure S3C), probably via inhibition of Bim_S_ (Figure S7B).

**Figure 6:**
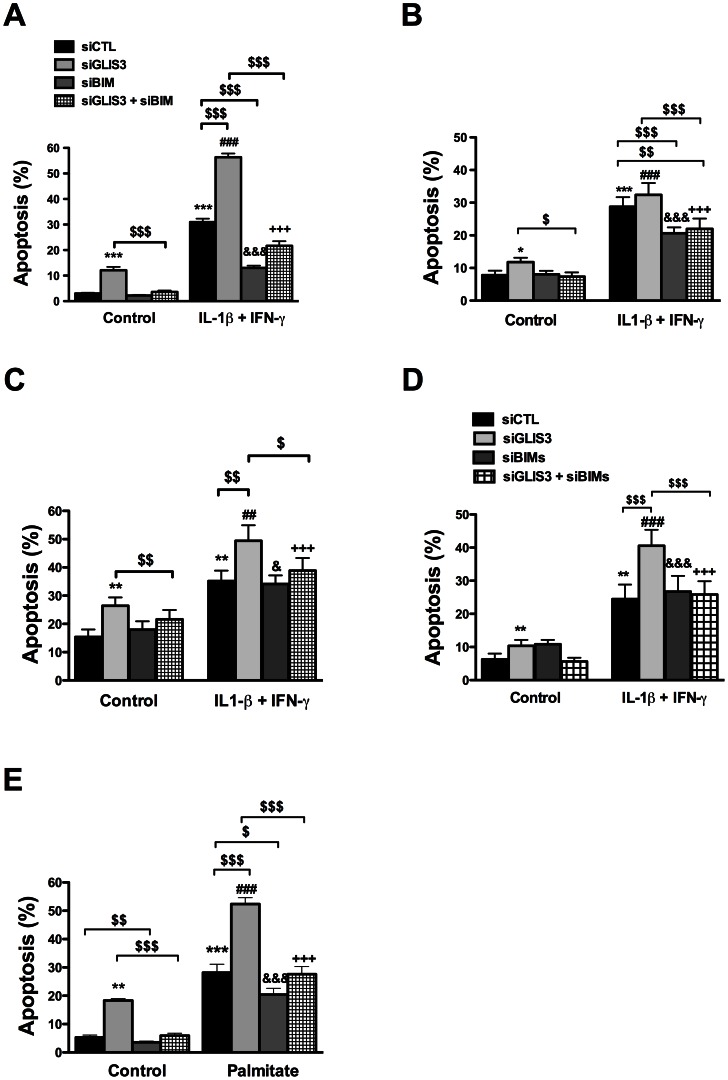
*Bim* mediates the potentiation of apoptosis in *GLIS3-*deficient cells. INS-1E cells (A, D and E), primary rat beta cells (B) and human dispersed islet cells (C) transfected with control or GLIS3 siRNAs were exposed to cytokines (A, B, C and D) or palmitate (E) for 24 h. (A, B, C and E) cells transfected with Bim siRNA (siBim); (D) cells transfected with a second Bim siRNA affecting preferentially Bim_S_ (siBim_S_). Apoptosis was then measured using nuclear dyes. Results are means ± SEM (n = 4–11). * *P*<0.05, ** *P*<0.01 or *** *P*<0.001 *vs*. siCTL without cytokines; ^##^ *P*<0.01 or ^###^ *P*<0.001 *vs*. siGLIS3; ^&^ *P*<0.05 or ^&&&^ *P*<0.001 *vs*. siBim; ^+++^ *P*<0.001 *vs*. siGLIS3 + siBim; ^$^ *P*<0.05, ^$$^ *P*<0.01 or ^$$$^ *P*<0.001 as indicated by the bars. ANOVA followed by paired *t*-test with Bonferroni's correction.

We have previously shown that this Bim siRNA markedly decreases expression of the three splice variants of *Bim* in cytokine-treated INS-1E cells [30]. In both INS-1E cells, primary beta cells and human islet cells *Bim* depletion by >50% (*P*<0.05) (Figure S6A, S6B and data not shown) abrogated the basal increase in apoptosis observed following *GLIS3* KD (Figure 6A, 6B and 6C). Interestingly, while Bim depletion protected human islet cells against apoptosis (Figure 6C), it failed to prevent the decrease in insulin secretion secondary to GLIS3 KD (data not shown), indicating dissociation between the functional and pro-apoptotic effects of GLIS3 KD. *Bim* KD also partially prevented the increase in cell death induced by *GLIS3* KD + cytokines (Figure 6A, 6B and 6C). These observations were confirmed with a second siRNA (Figure 6D) that induced a preferential inhibition of *Bim_S_* (71±4% inhibition of *Bim_S_*, *P*<0.001). To examine whether this beneficial effect of *Bim* KD was restricted to cytokines, we performed double KD for *GLIS3* and *Bim* and then exposed the cells to palmitate (Figure 6E). Palmitate treatment also preferentially increased expression of *Bim_S_* in INS-1E cells (Figure 5G, Figure S5G and S5H). *Bim* KD had only a minor protective effect against palmitate alone, in agreement with recent data suggesting that *DP5* and *PUMA* are the main mediators of palmitate-induced beta cell apoptosis [33], but it abrogated the additive effect of *GLIS3* KD upon palmitate exposure, decreasing cell death to the levels observed with palmitate alone (Figure 6E).

**Figure 7:**
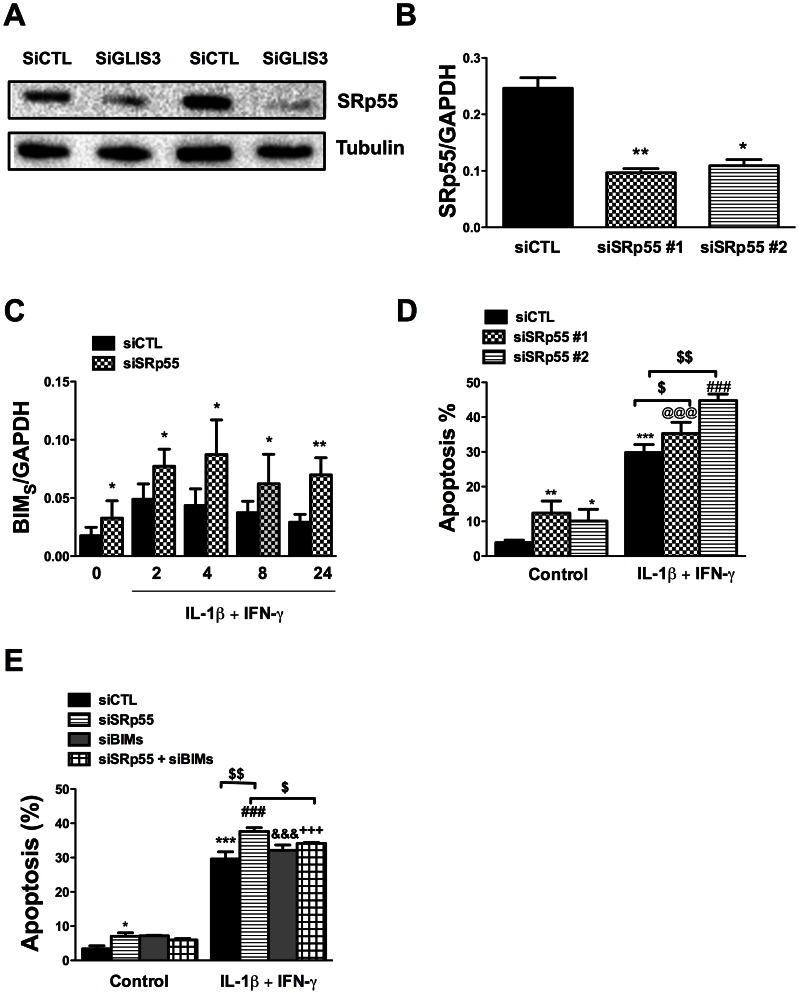
*GLIS3* KD decreases SRp55 expression in INS-1E cells. INS-1E cells were transfected with control, GLIS3 (A), SRp55 (B, C, D and E) and Bim*_S_* siRNAs (E). After 48 h cells were incubated with cytokines (C–E) for measuring apoptosis and collected at different time points for real-time PCR analyses. (A) Representative blot of 2 independent experiments for SRp55 protein expression after GLIS3 KD (n = 7; densitometry is provided in Results); (B) *SRp55* mRNA expression after KD with two different siRNAs (SRp55#1 and SRp55#2); (C) mRNA expression of *Bim_S_* after *SRp55* KD and exposure to cytokines; (D, E) apoptosis in cells transfected with SRp55 and/or a Bim siRNA targeting preferentially Bim_S_ (siBim_S_). Apoptosis was measured using nuclear dyes. Results are means ± SEM (n = 4–7). * *P*<0.05, ** *P*<0.01, *** *P*<0.001 *vs*. siCTL without cytokines ^@@@^ *P*<0.001 *vs*. siSRp55#1; ^###^ *P*<0.001 *vs*. siSRp55#2; ^&&&^ *P*<0.001 *vs*. siBim_S_; ^+++^ *P*<0.001 *vs*. siSRp55 + siBim_S_; ^$^ *P*<0.05 or ^$$^ *P*<0.01 as indicated by the bars. Paired t- test (7B and 7C) or ANOVA followed by paired *t*-test with Bonferroni's correction (7D and 7E).

To address the mechanisms by which GLIS3 affect Bim splicing, we examined the potential role of Pnn and SRp55, two splicing factors described in other tissues as potential regulators of Bim splicing [34], [35] and detected as present and modified by cytokines in human islets exposed to cytokines [21]. Pnn expression was not modified by GLIS3 KD (data not shown). On the other hand, GLIS3 KD decreased protein expression of SRp55 in INS-1E cells (Figure 7A) (43%±8% inhibition of SRp55 protein expression, p<0.05, n = 7), while GLIS3 overexpression augmented SRp55 expression basally and following cytokine exposure (Figure S7B). To assess the functional impact of decreased expression of SRp55, we inhibited it with two specific siRNAs (Figure 7B). After KD of SRp55, there was a significant increase of *Bim_S_* expression under both basal condition and following cytokine treatment (Figure 7C). We next evaluated whether SRp55 KD affects beta cell viability and observed an increase in apoptosis under basal condition and following cytokine exposure (Figure 7D) indicating a relevant role of SRp55 in viability. Double KD of SRp55 and Bim_S_ (71%±4% inhibition of *Bim_S_*, p<0.001) counteracted the increase in apoptosis caused by SRp55 KD (Figure 7D), suggesting a role for this splicing regulator in the downstream effects of GLIS3 (Figure 7E).

**Figure 8:**
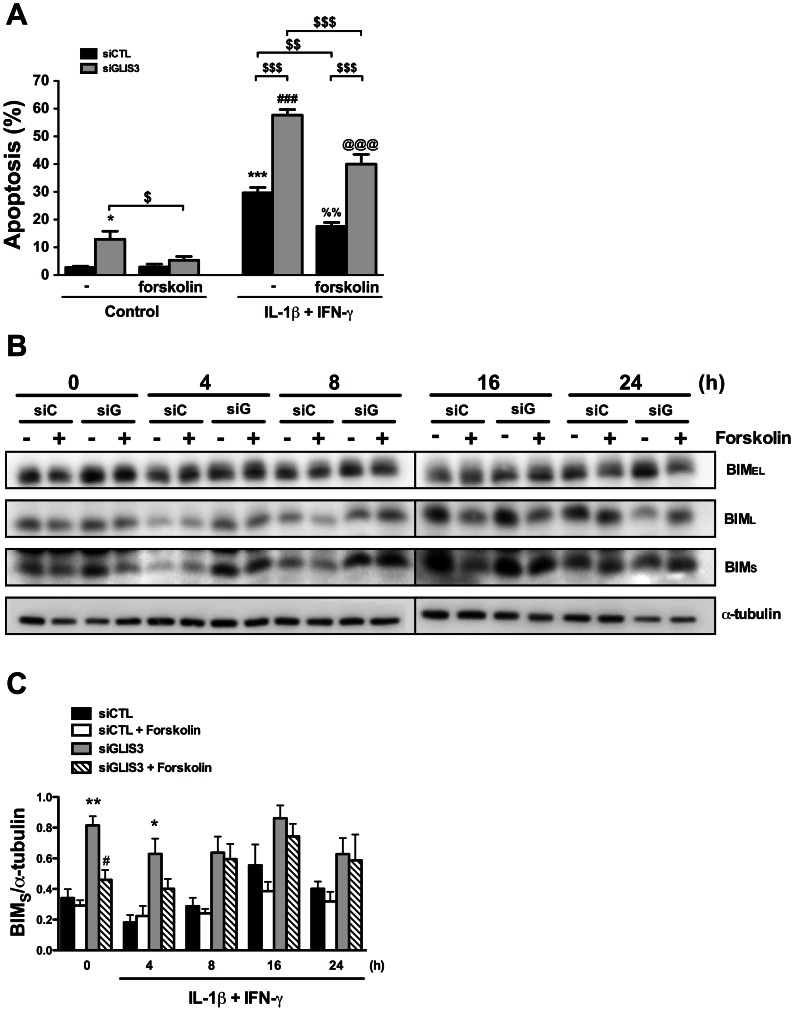
Forskolin partially prevents apoptosis following *GLIS3* KD. INS-1E cells transfected with control or GLIS3 siRNA were exposed to forskolin (20 µM) and/or cytokines for 24 h. After this period apoptosis was measured using nuclear dyes. (A) Apoptosis induced by cytokine treatment after siRNA transfection and forskolin exposure. Results are means ± SEM (n = 6) * *P*<0.05 or *** *P*<0.001 *vs*. siCTL without forskolin; ^###^ *P*<0.001 *vs*. siGLIS3 without forskolin; ^%%^ *P*<0.01 *vs*. siCTL with forskolin; ^@@@^ *P*<0.001 *vs*. siGLIS3 with forskolin; ^$^ *P*<0.05, ^$$^ *P*<0.01, ^$$$^ *P*<0.001 as indicated by the bars. ANOVA followed by paired *t*-test with Bonferroni's correction; (B) Representative blot of Bim_EL_, Bim_L_ and Bim_S_ protein isoform expression (n = 6); (C) Densitometry of Bim_S_ normalized by the housekeeping protein α-tubulin. Results are means ± SEM (n = 6) * *P*<0.05 or ** *P*<0.01 *vs*. siCTL, ^#^ *P*<0.05 *vs*. siGLIS3 by paired *t*-test.

cAMP generators have been previously shown to protect beta cells against both cytokine- and palmitate-induced apoptosis [36]–[39], and we evaluated whether forskolin could prevent beta cell apoptosis following *GLIS3* KD. Interestingly, forskolin nearly completely prevented the basal increase in apoptosis following *GLIS3* KD (Figure 8A), which was accompanied by a significant decrease in the expression of Bim_S_ but not Bim_EL_ or Bim_L_ (Figure 8B and 8C). In cytokine-treated GLIS3 KD deficient cells forskolin induced only a mild and partial protection, which was paralleled by a progressive restoration of Bim_S_ expression (Figure 8B and 8C).

## Discussion

Genome-wide association studies have allowed the identification of a large number of associations between specific loci and T1D or T2D. The mechanisms by which most of these candidate genes predispose to diabetes remain to be clarified. This emphasizes the need for detailed studies on the function of candidate genes in the key tissues involved in the development of diabetes. Taking into account the central role for beta cell failure in both T1D and T2D [13], it is of particular relevance to clarify the potential impact of these “diabetes genes” on pancreatic beta cell dysfunction and death.

There is little convincing genetic link between T1D and T2D to date [40]–[42], with the possible exception of Latent Autoimmune Diabetes in Adult (LADA), a particular form of diabetes that has been reported to share some susceptibility risk factors from both T1D and T2D [43]. To our knowledge the *GLIS3* locus is the only one showing association with genome-wide significance for both T1D, T2D or glucose metabolism traits in non-diabetic subjects, adults or children and adolescents, and in population-based cohorts [3]–[8]. *GLIS3* is the single gene located within the confidence interval of the region of association with T1D [3], and the SNPs that have been reported to be associated with T1D, T2D and T2D-related traits are all in very strong linkage disequilibrium (LD) to each other (pairwise correlation coefficient r^2^ of 0.95 to 1.0 between the strongest associated SNPs for the key studies [3], [4], [6], [7], [44]), supporting the hypothesis that a unique variant near *GLIS3* may be responsible for all the reported associations with these common diabetes and related traits. Furthermore, a review of all the published genetic studies and available data on T1D, T2D and T2D-related traits indicated that the orientation of association is concordant between all these traits (C. Julier, unpublished observations), with the same allele associated with increased risk of T1D, increased risk of T2D, increased fasting glucose, decreased fasting insulin level, decreased HOMA-B and glucose stimulated insulin release (nominal *P*-values for association with these traits <10^−3^ [4], [5]), suggesting the role of a shared mechanism between both forms of diabetes.

Pancreatic islets from T2D patients have a nearly 50% decrease in *GLIS3* mRNA expression as compared to islets obtained from non-diabetic subjects (*P*<0.001; data re-calculated from [45] and confirmed by RT-PCR analysis of whole islets and FACS-purified human beta cells; Bugliani M, Marselli L and Marchetti P, unpublished data), but it remains to be determined whether this is a direct effect of the risk alleles on GLIS3 expression or secondary to chronic exposure to high glucose levels. Similarly, *GLIS3* was found to be one of the most differentially expressed genes between beta cells from T2D and non-diabetic subjects [46]. The fact that recessive loss-of-function mutations in *GLIS3* cause severe neonatal diabetes in humans [1] and in transgenic mouse models [9], [10], secondary to a major decrease in beta cell mass, suggests that this transcription factor is necessary for beta cell development and differentiation. Together, these genetic and functional observations indicate that *GLIS3* itself is the susceptibility gene responsible for the observed associations with T1D, T2D and T2D-related traits. The region strongly associated with T1D as defined by Barrett *et al.* [3] maps to the 5′ region of the *GLIS3* long transcript, which is pancreas and thyroid specific [1] and includes the first exons and corresponding promoter region. Of note, all the SNPs in LD with diabetes and associated SNPs are non-coding. This suggests that the responsible variant affects the regulation of *GLIS3* expression in pancreatic beta cells, most likely through a reduction of *GLIS3* expression predisposing to T1D and T2D. It is thus important to understand whether these milder phenotypes affect the resistance of adult beta cells to challenges provided by immune-, viral- or metabolic-mediated stress. These stresses may cross talk with candidate genes for T1D and T2D.

Our present observations suggest that a relatively mild reduction of *GLIS3* gene expression in beta cells by two independent siRNAs decreases expression of *Pdx1*, *MafA*, *Ins2* and *Glut2* and inhibit glucose oxidation and glucose-induced insulin secretion. These findings are in line with evidence obtained in foetal, neonatal or adult mouse beta cells [9], [11], and suggest a key role for *GLIS3* in maintaining the beta cell differentiated phenotype.

Of particular interest in the context of diabetes is the observation that *GLIS3* KD increases rat beta cell apoptosis under basal condition and sensitizes the cells to death induced by pro-inflammatory cytokines (IL-1β + IFN-γ), the viral by-product dsRNA, and the free fatty acids oleate and palmitate, while *GLIS3* up-regulation protects against cytokine-induced apoptosis (present data). *GLIS3* KD also increases apoptosis of human islet cells under both basal condition and following exposure to IL-1β + IFN-γ. This broad range of sensitization to pro-apoptotic stimuli by *GLIS3* KD suggests that *GLIS3*, besides contributing to maintain beta cell function, provides signals required for preservation of cell viability. In line with these observations, suppression of *Pdx1*, a key transcription factor for the maintenance of the differentiated phenotype of beta cells, triggers beta cell death via dissipation of the mitochondrial inner membrane electrochemical gradient Deltapsi(m) [47]. *GLIS3* KD also contributes to beta cell apoptosis via a mitochondrial phenomenon, namely triggering of the intrinsic pathway of apoptosis as a result of the activation of the BH3-only protein Bim (see below). Decreased *Pdx1* expression sensitizes pancreatic beta cells to ER stress [48], but this is not the case for *GLIS3* KD, as indicated by normal expression of *Chop* (present findings) and other ER stress markers (data not shown).

The increase in cell death in *GLIS3* deficient cells is secondary to activation of the intrinsic pathway of apoptosis, as indicated by Cytochrome *c* release to the cytosol, Bax translocation to the mitochondria and activation of caspases 9 and 3. A detailed analysis of the upstream pathways implicated in *GLIS3* KD-induced beta cell apoptosis indicated modulation of alternative splicing of the pro-apoptotic BH3-only protein Bim, favouring expression of the most pro-apoptotic splice variant of *Bim*, namely *Bim_S_* [49], [50]. In agreement with these observations, *Bim* depletion abrogated the pro-apoptotic effects of *GLIS3* KD alone or in combination with pro-inflammatory cytokines or palmitate. Bim can bind to and inhibit most anti-apoptotic Bcl-2 proteins, besides directly activating the pro-apoptotic protein Bax [51]. Importantly, *Bim* contributes to cytokine- [20], [30], virus- [23] and high glucose-induced [52] pancreatic beta cell apoptosis. Previous observations in pancreatic beta cells indicated that *Bim* can be regulated by cytokines at the transcriptional [30], [53] or phosphorylation [20] level. The present study is the first to show regulation of *Bim* function in beta cells by changes in splicing. There are three main isoforms of *Bim*, namely *Bim_EL_*, *Bim_L_*, and *Bim_S_* that are generated by alternative splicing [32]. *Bim_EL_* and *Bim_L_* have a binding site for the dynein light chain 1 which decreases their pro-apoptotic activity via sequestration to the cytoskeleton [32], [54], while *Bim_S_* is free to exert its potent pro-apoptotic activity [49], [50].

Alternative splicing affects more than 90% of human genes [55]. It generates enormous proteome diversity, and may have a major impact on cell survival, exposure of novel antigenic epitopes, alteration of surface location of antigens and posttranslational modifications. There is a growing interest in the role of alternative splicing in several autoimmune diseases [56], but nearly nothing is known on its role in pancreatic beta cell dysfunction and death in diabetes. We have recently shown that beta cell exposure to pro-inflammatory cytokines modifies alternative splicing of hundreds of expressed genes and affects expression of more than 50 splicing-regulating proteins [21], [57]. Palmitate also modifies alternative splicing of a different group of genes in human islets (Cnop M, Sammeth M, Bottu G and Eizirik DL, unpublished data). The present observations provide the first indication that a candidate gene for diabetes may act by regulating alternative splicing. This effect of GLIS3 KD is mediated, at least in part, via down regulation of the splicing factor SRp55 (Figure 7). This was confirmed by the reverse experiment, i.e. GLIS3 overexpression induced SRp55 and prevented Bim_S_ production (Figure S7B). In line with this, the inhibition of SRp55 led to an increase in *Bim_S_* expression and beta cell apoptosis (Figure 7). These results suggest that GLIS3 regulates the expression of splicing factors and consequently the splicing of their target genes. It remains to be clarified whether this is a direct effect or a secondary phenomenon via downstream regulation of other genes.

In conclusion, the present observations suggest that modifications in expression of the candidate gene *GLIS3* may contribute to both T1D and T2D by favouring beta cell apoptosis. This takes place to a large extent via modified alternative splicing of the pro-apoptotic protein Bim. Additional studies are now required to characterize this new avenue for functional studies on candidate genes for diabetes, namely their cross-talk with alternative splicing and other processes regulating generation of gene/protein diversity.

## Materials and Methods

### Ethics statement

Human islet collection and handling were approved by the local Ethical Committee in Pisa, Italy. Wistar rats were used according to the rules of the Belgian Regulations for Animal Care with approval of the Ethical Committee for Animal Experiments of the ULB.

### Culture of INS-1E cells, FACS-purified rat beta cells, and human islet cells

INS-1E cells (kindly provided by C. Wollheim, Centre Medical Universitaire, Geneva, Switzerland) at passages 60–72 were cultured in RPMI 1640 GlutaMAX-I medium, supplemented with 5% heat-inactivated foetal bovine serum (FBS), 50 units/ml penicillin, 50 µg/ml streptomycin, 10 mM HEPES, 1 mM Na-pyruvate, and 50 µM 2-mercaptoethanol in a humidified atmosphere at 37°C and 5% CO_2_.

Isolated pancreatic islets of male Wistar rats (Charles River Laboratories, Brussels, Belgium), housed following the guidelines of Belgian Regulations for Animal Care, were dispersed and beta cells purified by autofluorescence-activated cell sorting (FACSAria, BD Bioscience, San Jose, CA, USA) [58], [59]. Beta cells (93±2% purity; n = 6) were cultured in Ham's F-10 medium containing 10 mM glucose, 2 mM glutamine, 50 µM 3-isobutyl-L-methylxanthine, 0.5% fatty acid-free bovine serum albumin (BSA) (Roche, Indianapolis, IN, USA), 5% FBS, 50 units/ml penicillin, and 50 µg/ml streptomycin [59]. The same medium but without FBS was used during cytokine exposure.

Human islet cells from 8 non-diabetic donors (age 66±5 years, five men/three women, body mass index 25.7±0.9 Kg/m^2^) were isolated in Pisa, with the approval of the Ethics Committee of the University of Pisa. Islets were isolated by enzymatic digestion, and density-gradient purification [60]. They were then cultured in M199 medium containing 5.5 mM glucose and shipped to Brussels, Belgium within 1–5 days of isolation. After overnight recovery in Ham's F-10 containing 6.1 mM glucose, 10% FBS, 2 mM GlutaMAX, 50 µM 3-isobutyl-1-methylxanthine, 1% BSA, 50 U/ml penicillin and 50 µg/ml streptomycin, islets were dispersed, transfected with siCTL, siGLIS3, siBim or siGLIS3/siBim and exposed or not to cytokines for 24 h. The same medium but without FBS was used during cytokine exposure. The percentage of beta cells in the dispersed islet preparations, as determined by immunohistochemistry for insulin [37], was 48±6%.

### RNA interference

The siRNAs used in the study are described in Table S1. The optimal concentration of siRNA used for cell transfection (30 nM) was established previously [61]. Cells were transfected using the Lipofectamine RNAiMAX lipid reagent (Invitrogen, Carlsbad, CA, USA) as previously described [31]. Allstars Negative Control siRNA (Qiagen, Venlo, the Netherlands) was used as negative control (siCTL). siCTL does not affect beta cell gene expression or insulin release, as compared with nontransfected cells [31], [61], [62]. Beta cells transfected with siRNAs were used for experiments 24–48 h after transfection.

### Generation of recombinant adenovirus and cell infection

To express *GLIS3* in insulin-secreting cells, we obtained from SIRION Biotech (Munich, Germany) a recombinant adenovirus comprising fragments of the mouse *GLIS3* mRNA (GenBank: NM_175459).

The murine *GLIS3* coding region was amplified by PCR from cDNA clone BC167165 purchased from Source Bioscience (Berlin, Germany) and was cloned via Nhe1 and EcoRV into the shuttle vector pO6-A5-CMV to give pO6-A5-CMV-GLIS3. The CMV-GLIS3-SV40-pA region of pO6-A5-CMV-GLIS3 was then transferred via recombination in a BAC vector containing the genome of a replication deficient Ad5-based vector deleted in E1/E3 genes. Presence and correctness of the GLIS3-ORF in the resulting BAC-vector BA5-CMV-GLIS3 was confirmed by DNA-sequencing.

An adenovirus expressing the luciferase protein (Ad-LUC) was used as control [63]. INS-1E cells were infected as previously described [63].

### Cell treatment

The cytokine concentrations used were based on previous dose-response experiments performed by our group [64], [65] and were 10 units/ml or 50 units/ml of recombinant human IL-1β for INS-1E cells or primary rat beta cells/human islet cells, respectively (a kind gift from Dr. C.W. Reinolds, National Cancer Institute, Bethesda, MD-USA) and 100 units/ml or 500 units/ml of recombinant rat IFN-γ for INS-1E cells and primary rat beta cells or 1000 units/ml of recombinant human IFN-γ for human islet cells (R&D Systems, Abingdon, UK). Culture supernatants from cytokine-treated cells were collected for nitrite determination (nitrite is a stable product of NO oxidation) at OD540 nm using the Griess method. The synthetic dsRNA polyinosinic-polycytidylic acid (PIC; Sigma, St Louis, LO, USA) was used at the final concentration of 1 µg/ml [19]. Cellular transfection with PIC was made as described for siRNA, with the difference that Lipofectamine 2000 was used instead of Lipofectamine RNAiMAX [19]. Oleate and palmitate (sodium salt, Sigma, Bornem, Belgium) were dissolved in 90% (vol./vol.) ethanol and diluted 1∶100 to a final concentration of 0.5 mM in the presence of 1% charcoal-absorbed BSA, corresponding to a free fatty acid/BSA ratio of 3.4 [66], [67]. Forskolin was diluted in DMSO and used at final concentration of 20 µM (Sigma).

### mRNA extraction and real-time PCR

mRNA was extracted and reverse transcribed as described [59]. Expression of target genes was determined by real-time PCR using SYBR Green [59], [68] and comparison with a standard curve [69]. Expression values were corrected by the housekeeping gene glyceraldehyde-3-phosphate dehydrogenase (*GAPDH*) for INS-1E and primary rat beta cells and *β-actin* for human islet cells. *GAPDH* or *β-actin* expression is not modified under the present experimental conditions [18], [66], [70]. Primer sequences are described in Table S2. Primers for *MafA*, *Pdx1*, *INS2*, *Glut2*, *Chop*, *Dp5* and *Puma* were described previously [53], [71].

### Glucose oxidation and insulin secretion

D-[U-^14^C] glucose (specific activity: 300 mCi/mM, concentration: 1 mCi/ml, Perkin Elmer, Waltham, MA, USA) was used to evaluate glucose oxidation in control and *GLIS3* KD cells exposed to different glucose concentrations as described [72]. The rate of glucose oxidation was expressed as pmol/120 min.10^5^ cells.

For determination of insulin secretion, INS-1E cells were incubated for 1 h in glucose-free RPMI GlutaMAX-I medium and then incubated for 30 min in Krebs-Ringer solution. Cells were then exposed to 1 mM, 10 mM or 10 mM glucose with forskolin (20 µM) for 30 min. Insulin was measured in the supernatant by the rat insulin ELISA kit (Mercodia, Uppsala, Sweden). Results were normalized by the insulin content measured after cell lyses. Insulin accumulation in the medium of cultured human islets was measured by the human insulin ELISA kit (Mercodia, Uppsala, Sweden).

### Assessment of cell viability

The percentage of viable, apoptotic and necrotic cells was determined following 15 min of incubation with 5 mg/ml of the DNA-binding dyes propidium iodide (PI, Sigma) and Hoechst 33342 (HO, Sigma). This method is quantitative and has been validated for use in pancreatic beta cells and INS-1E cells by comparison with electron microscopy, caspase-3 activation and DNA laddering [18], [59], [66], [73], [74]. A minimum of 600 cells was counted in each experimental condition. Viability was evaluated by two independent observers, one of them unaware of sample identity. The agreement between findings obtained by the two observers was >90%. In some experiments apoptosis was confirmed by Western blot analysis of cleaved caspase-9 and -3, cytoplasmic cytochrome *c* release and BAX translocation to the mitochondria.

### Western blot and assessment of cytochrome c release

INS-1E cells were lysed in Laemmli buffer and equal amounts of total protein were heated at 100°C for 5 min, resolved by electrophoresis in 10–14% SDS-polyacrylamide gel and electro-blotted onto nitrocellulose membranes. Immunodetection was performed after overnight incubation with antibodies for cleaved caspase 9 and 3 (Cell Signaling, Danvers, USA), Bcl-2 (Cell Signaling, Danvers, USA), Bcl-xL antibody (Cell Signaling, Danvers, USA), Bim and p-Bim antibodies (Cell Signaling, Danvers, USA), SRp55 antibody (LifeSpan Biosciences), STAT1 and p-STAT1 antibodies (Cell Signaling). α-tubulin (Cell Signaling) was used as the loading control. Membranes were then exposed to 150 ng/ml secondary peroxidase-conjugated antibody (anti IgG (H+L)-HRP, Invitrogen) for 2 h at room temperature and visualized by chemiluminescence (SuperSignal, Pierce Biotechnology, Rockford, IL, USA). Bands were detected by a LAS-3000 CCD camera (Fujifilm, Tokyo, Japan). The densitometry of the bands was evaluated using the Aida Analysis software (Raytest, Straubenhardt, Germany).

For the assessment of cytochrome *c* release, INS-1E cells harvested in cold PBS were centrifuged (500 g for 2 min) and resuspended with 50 µl lysis buffer (75 mM NaCl, 1 mM NaH_2_PO_4_, 8 mM Na_2_PO_4_, 250 mM sucrose, 21 µg/µl aprotinin, 1 mM PMSF and 0.8 µg/µl digitonin) and vortexed for 30 s. After centrifugation (20,000 g for 1 min) the supernatant was collected as the cytoplasmic fraction. The pellet was resuspended in 50 µl lysis buffer containing 8 µg/µl digitonin, centrifuged (1 min at 20,000 g) and the supernatant collected as the mitochondrial fraction [23], [37]. Equal amounts of proteins were used for Western-blotting with antibodies for cytochrome *c* (BD Biosciences) (cytoplasmic protein), apoptosis-inducing factor (AIF) and cytochrome *c* oxidase (COX IV) (mitochondrial proteins) (Cell Signaling). β-actin was used as the loading control.

### Immunofluorescence

INS-1E cells were plated on polylysine-coated glass culture slides (BD Biosciences). After transfection and treatment, cells were fixed for 15 min in 4% paraformaldehyde, washed with PBS and permeabilized in Triton X-100 0.1% for 5 min. Slides were then blocked using 5% goat serum and incubated overnight at 4°C with a Bax antibody (Santa Cruz Biotechnology) plus ATP synthase β antibody (mitochondrial marker) (BD Biosciences). Cells were washed with PBS and incubated for 1 h with the appropriate Alexa fluor 488 or 555-conjugated antibodies (Invitrogen). Cells were stained with Hoechst 33342, mounted and photographed using fluorescence microscopy (Axio Imager, Carl Zeiss, Zaventem, Belgium) [62].

### Statistics

Data are presented as mean ± SEM. Comparisons were performed by two-tailed paired *t*-test or by ANOVA followed by paired *t*-test with Bonferroni correction, as adequate.

A *P* value<0.05 was considered as statistically significant.

## Supporting Information

Figure S1 Confirmation of the effects of *GLIS3* KD using different siRNAs. INS-1E cells and human islet cells were transfected with siCTL and different siRNAs for GLIS3 (#2) and then exposed or not to cytokines. After 24 h cells were used for real-time PCR analyses and apoptosis was measured. (A, D) Confirmation of *GLIS3* KD using a second siRNA for *GLIS3* in INS-1E cells (A) and human islet cells (D); (B) mRNA expression of *INS2* after *GLIS3* KD in INS-1E cells; (C, E) apoptosis induced by cytokine treatment of INS-1E cells or human islet cells after *GLIS3* KD. Results are means ± SEM (n = 4) * *P*<0.05, ** *P*<0.01 or *** *P*<0.001 *vs*. siCTL by paired *t*-test.
(TIF)

Figure S2 *GLIS3* KD induces caspase-9 and -3 cleavage and Cytochrome *c* release. INS-1E cells were transfected with siCTL and two different siRNAs for *GLIS3* and then exposed or not to cytokines for 24 h. (A, B) Densitometry of Western blots for cleaved caspase-9 and -3, normalized by the housekeeping protein α-tubulin (a representative blot is shown in Figure 3A). (C) Densitometry analysis of the Western blots for cytochrome *c* release from the mitochondria (a representative blot is shown in Figure 3B). Results are means ± SEM (n = 4). * *P*<0.05, ** *P*<0.01 or *** *P*<0.001 *vs*. siCTL by paired *t*-test.
(TIF)

Figure S3 *GLIS3* overexpression induces MafA expression and prevents cytokine-induced apoptosis. INS-1E cells were infected or not (NI, non-infected) with an adenoviral vector encoding *GLIS3* (AdGLIS3) at MOIs ranging from 0.1 to 50, and then exposed or not to cytokines for 24 h. (A) Confirmation of *GLIS3* mRNA overexpression by RT-PCR 24 h after infection. Results are means ± SEM (n = 4). * *P*<0.05 and ** *P*<0.01 *vs*. NI by paired *t*-test. (B) Representative blot of 3 independent experiments for MafA protein expression after infection with AdGLIS3 for 24 or 48 h. (C) Apoptosis of INS-1E cells induced by a 24 h cytokine treatment after infection with AdLUC (control adenoviral vector) or AdGLIS3 at MOI 10 for 24 h. Results are means ± SEM (n = 4). *** *P*<0.001 *vs*. AdLUC without cytokines; ^###^ *P*<0.001 *vs*. AdGLIS3 without cytokines; ^$$$^ *P*<0.001 as indicated by the bars. ANOVA followed by paired *t*-test with Bonferroni's correction.
(TIF)

Figure S4 *GLIS3* KD does not increase nitric oxide production or *CHOP*, *DP5* and *PUMA* mRNA expression. INS-1E cells transfected with siCTL or siGLIS3 were exposed or not to cytokines and then used for nitrite measurement and real-time PCR. (A) Nitrite measurement (reflecting nitric oxide production) after *GLIS3* KD and 24 h of cytokine treatment; (B–D) mRNA expression of *CHOP*, *DP5* and *PUMA* after *GLIS3* KD and a time course of cytokine exposure. Results are means ± SEM corrected by the housekeeping gene *GAPDH* (n = 4) * *P*<0.05 or ** *P*<0.01 *vs*. siCTL. Paired *t*-test.
(TIF)

Figure S5 Impact of *GLIS3* KD on *Bim_EL_* and *Bim_L_* expression in basal condition and after cytokine or palmitate treatment. After 48 h of control or GLIS3 siRNA, INS-1E cells were incubated with cytokines (A–D) or palmitate (G–H) and collected at different time points for Western blot and real-time PCR analyses. (A, B) Densitometry of Bim_EL_ and Bim_L_ protein expression normalized by α-tubulin; (C, D, G and H) mRNA expression of *Bim_EL_* and *Bim_L_* normalized by the housekeeping gene *GAPDH*. (E and F) mRNA expression in human islets of *Bim_EL_* and *Bim_L_* normalized by the housekeeping gene *β-actin* after a 48 h of control or GLIS3 siRNA transfection. Results are means ± SEM (n = 4). * *P*<0.05, ** *P*<0.01 or *** *P*<0.001 *vs*. siCTL by paired *t*-test.
(TIF)

Figure S6 Double KD of *GLIS3* and *Bim* in primary rat beta cells. FACS-purified rat beta cells were transfected with control, GLIS3 or Bim siRNA. After 48 h cells were treated with cytokines for 24 h. (A, B) mRNA expression of *GLIS3* and *Bim_S_*. Results are means ± SEM corrected by the housekeeping gene *GAPDH* (n = 4). * *P*<0.05 *vs*. siCTL; ^$^ *P*<0.05 or ^$$$^ *P*<0.001 as indicated by the bars. ANOVA followed by paired *t*-test with Bonferroni's correction.
(TIF)

Figure S7 *GLIS3* overexpression down regulates Bim_S_ and decreases cytokine-induced cleavage of caspase 3. INS-1E cells were infected with AdLUC or AdGLIS3 at MOI 10 and 24 h later exposed or not to cytokines for an additional 24 h. (A) mRNA expression of *GLIS3* normalized by *GAPDH*. Results are means ± SEM (n = 4). (B) Representative blot of 2–4 independent experiments for Bim_S_, cleaved caspase-3 and SRp55 and the housekeeping protein α-tubulin. ^$^ *P*<0.05 as indicated by the bars. Paired *t*-test.
(TIF)

Table S1 Sequences of siRNAs used to KD gene/protein expression.
(DOC)

Table S2 Primer sequences and their respective PCR product lengths. ST denotes standard PCR, RT denotes real time qPCR.
(DOC)
