# Supplementary figures and images for: GLIS3, a Susceptibility Gene for Type 1 and Type 2 Diabetes, Modulates Pancreatic Beta Cell Apoptosis via Regulation of a Splice Variant of the BH3-Only Protein Bim
Source: PLoS Genet. 2013 May 30;9(5):e1003532. doi: 10.1371/journal.pgen.1003532 (PMC3667755; doi:10.1371/journal.pgen.1003532)

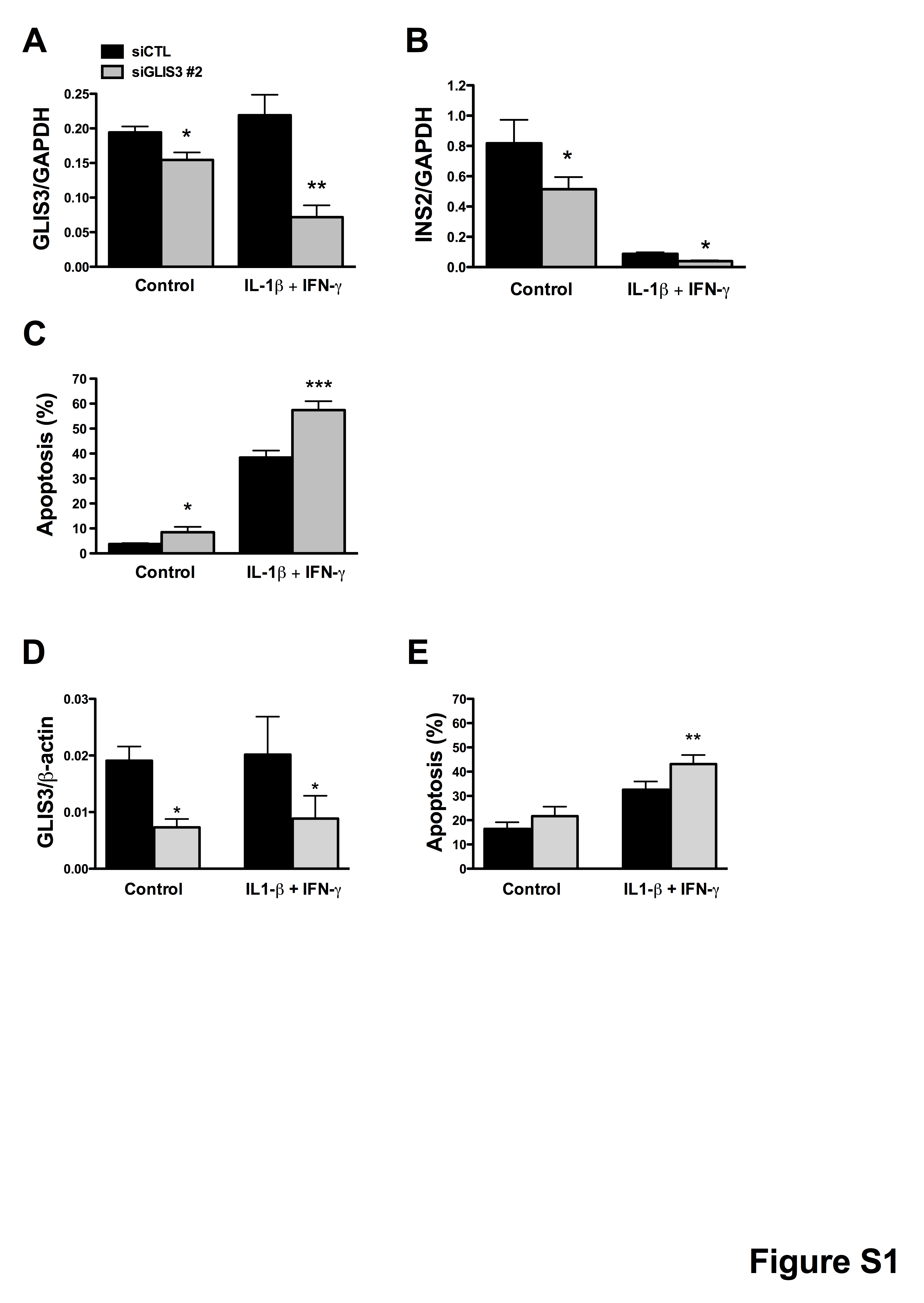

Supplement: Figure S1 — Confirmation of the effects of GLIS3 KD using different siRNAs. INS-1E cells and human islet cells were transfected with siCTL and different siRNAs for GLIS3 (#2) and then exposed or not to cytokines. After 24 h cells were used for real-time PCR analyses and apoptosis was measured. (A, D) Confirmation of GLIS3 KD using a second siRNA for GLIS3 in INS-1E cells (A) and human islet cells (D); (B) mRNA expression of INS2 after GLIS3 KD in INS-1E cells; (C, E) apoptosis induced by cytokine treatment of INS-1E cells or human islet cells after GLIS3 KD. Results are means ± SEM (n = 4) * P<0.05, ** P<0.01 or *** P<0.001 vs. siCTL by paired t-test. (TIF) [file pgen.1003532.s001.tif]

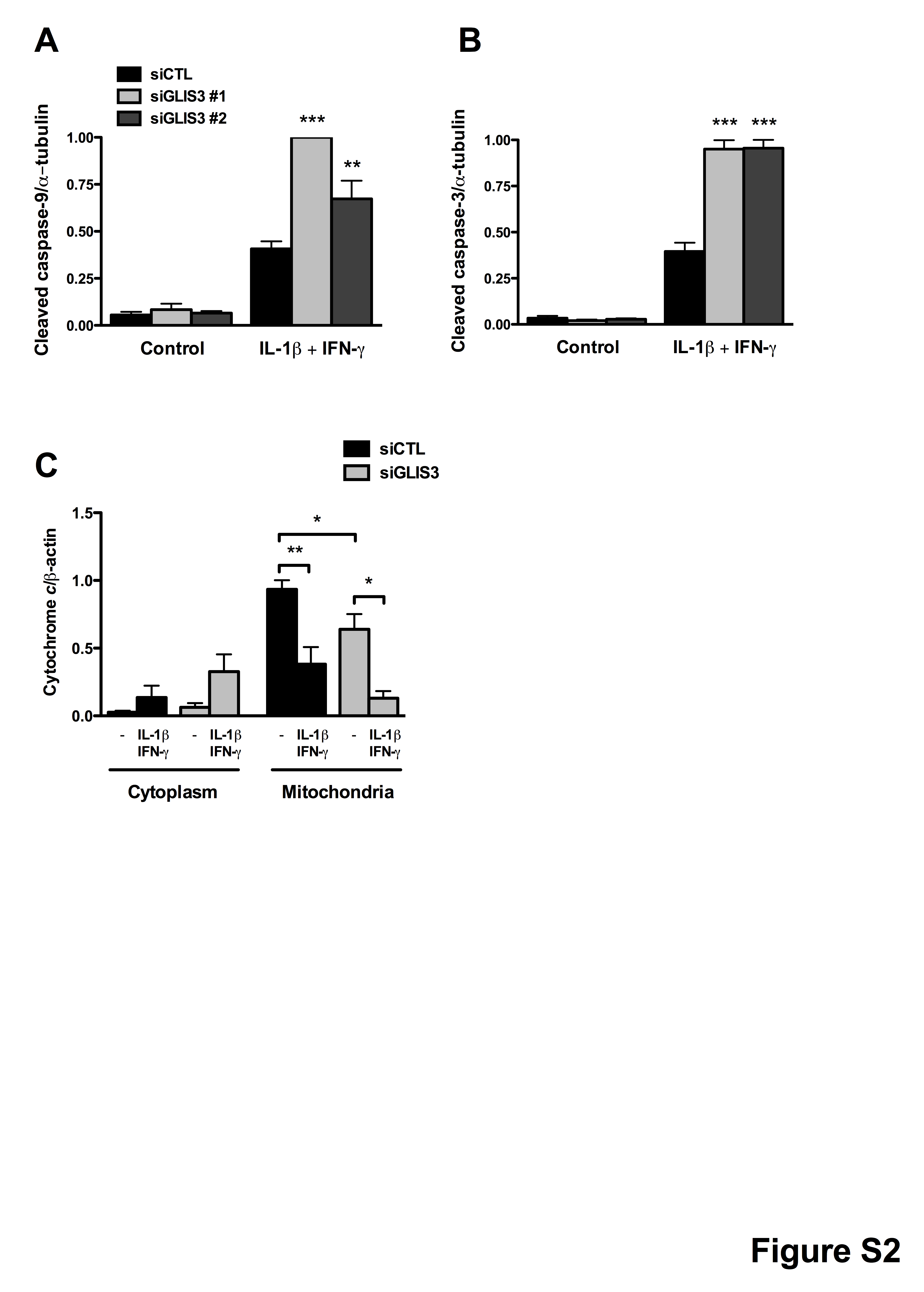

Supplement: Figure S2 — GLIS3 KD induces caspase-9 and -3 cleavage and Cytochrome c release. INS-1E cells were transfected with siCTL and two different siRNAs for GLIS3 and then exposed or not to cytokines for 24 h. (A, B) Densitometry of Western blots for cleaved caspase-9 and -3, normalized by the housekeeping protein α-tubulin (a representative blot is shown in Figure 3A). (C) Densitometry analysis of the Western blots for cytochrome c release from the mitochondria (a representative blot is shown in Figure 3B). Results are means ± SEM (n = 4). * P<0.05, ** P<0.01 or *** P<0.001 vs. siCTL by paired t-test. (TIF) [file pgen.1003532.s002.tif]

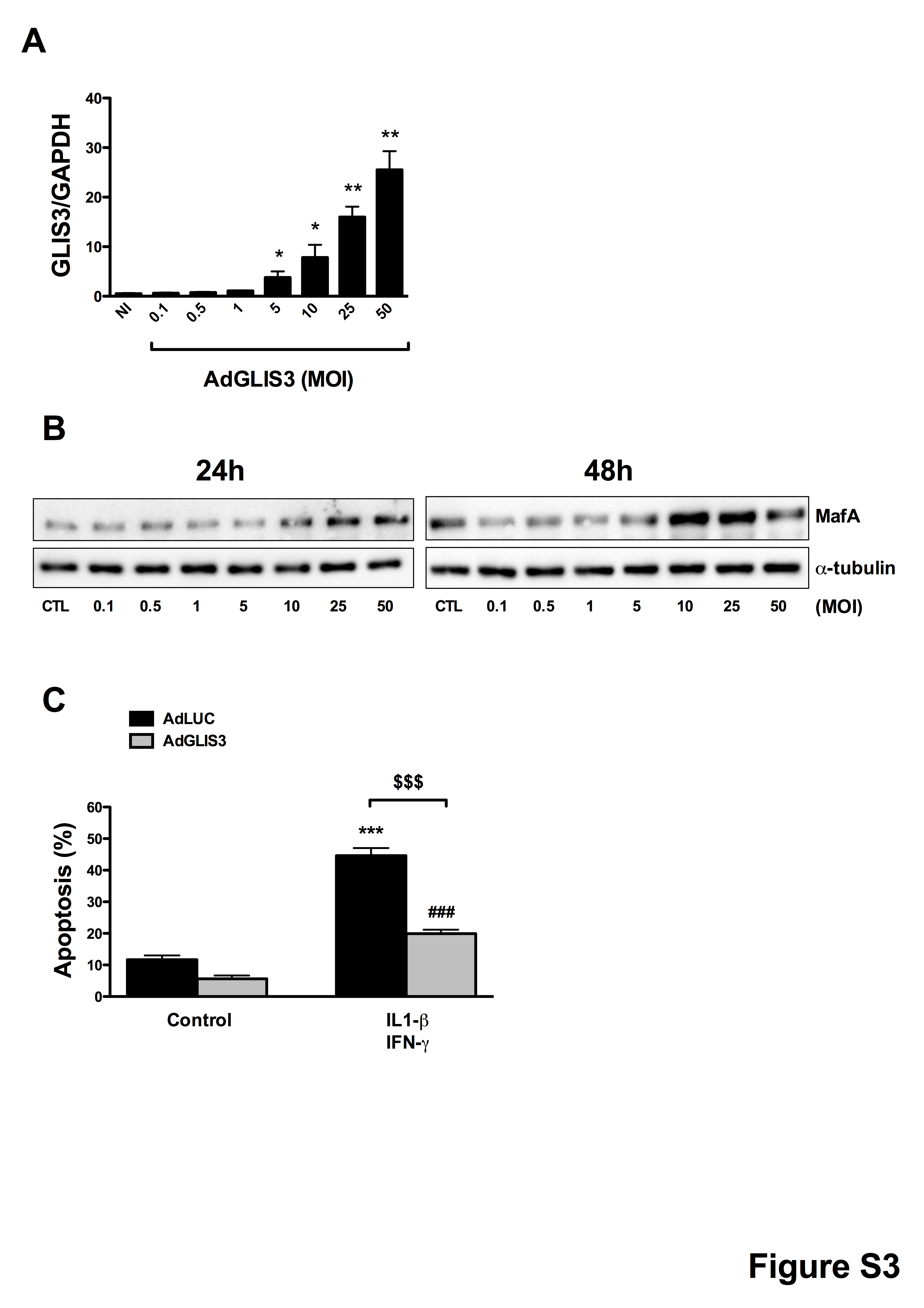

Supplement: Figure S3 — GLIS3 overexpression induces MafA expression and prevents cytokine-induced apoptosis. INS-1E cells were infected or not (NI, non-infected) with an adenoviral vector encoding GLIS3 (AdGLIS3) at MOIs ranging from 0.1 to 50, and then exposed or not to cytokines for 24 h. (A) Confirmation of GLIS3 mRNA overexpression by RT-PCR 24 h after infection. Results are means ± SEM (n = 4). * P<0.05 and ** P<0.01 vs. NI by paired t-test. (B) Representative blot of 3 independent experiments for MafA protein expression after infection with AdGLIS3 for 24 or 48 h. (C) Apoptosis of INS-1E cells induced by a 24 h cytokine treatment after infection with AdLUC (control adenoviral vector) or AdGLIS3 at MOI 10 for 24 h. Results are means ± SEM (n = 4). *** P<0.001 vs. AdLUC without cytokines; ### P<0.001 vs. AdGLIS3 without cytokines; $$$ P<0.001 as indicated by the bars. ANOVA followed by paired t-test with Bonferroni's correction. (TIF) [file pgen.1003532.s003.tif]

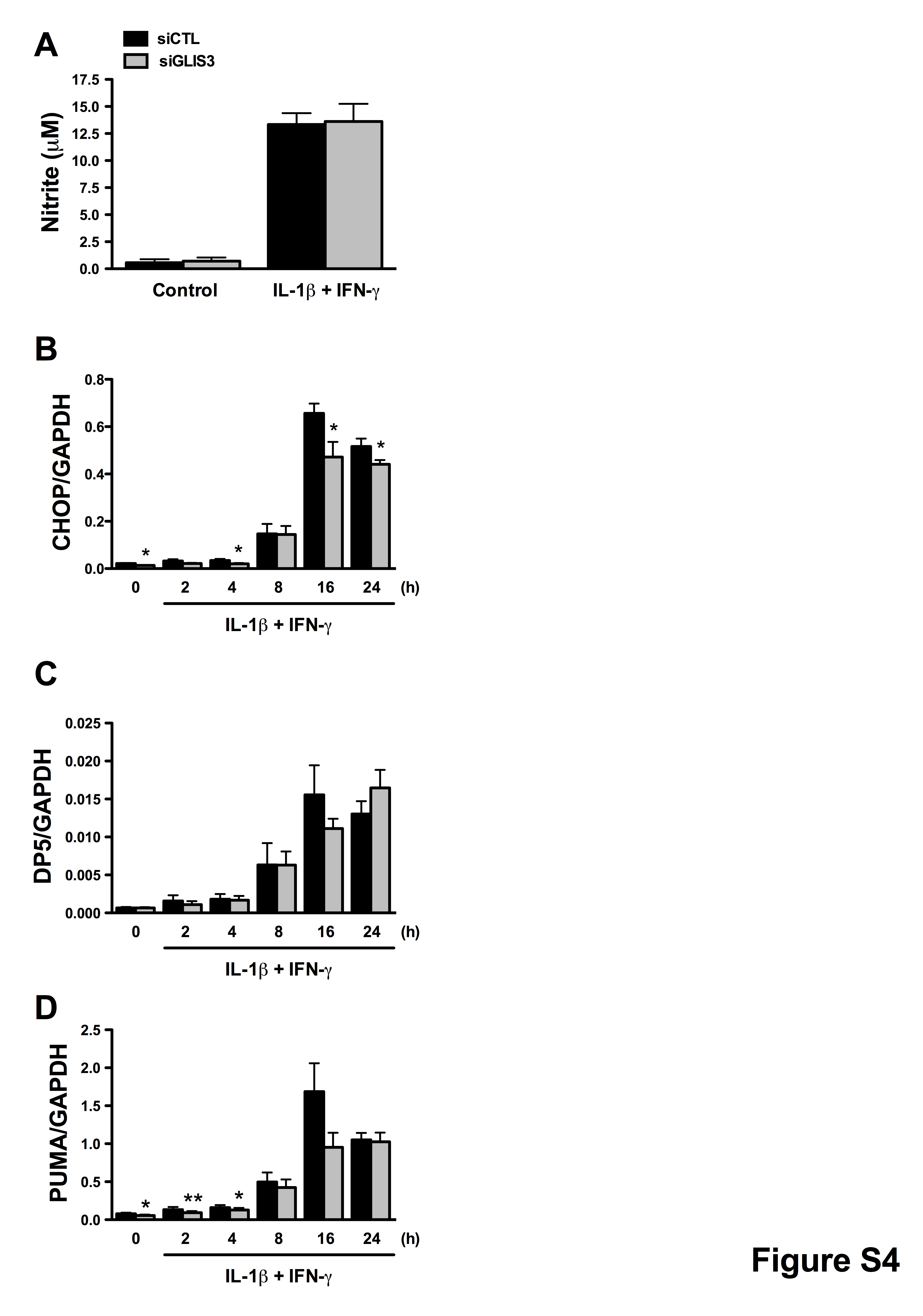

Supplement: Figure S4 — GLIS3 KD does not increase nitric oxide production or CHOP, DP5 and PUMA mRNA expression. INS-1E cells transfected with siCTL or siGLIS3 were exposed or not to cytokines and then used for nitrite measurement and real-time PCR. (A) Nitrite measurement (reflecting nitric oxide production) after GLIS3 KD and 24 h of cytokine treatment; (B–D) mRNA expression of CHOP, DP5 and PUMA after GLIS3 KD and a time course of cytokine exposure. Results are means ± SEM corrected by the housekeeping gene GAPDH (n = 4) * P<0.05 or ** P<0.01 vs. siCTL. Paired t-test. (TIF) [file pgen.1003532.s004.tif]

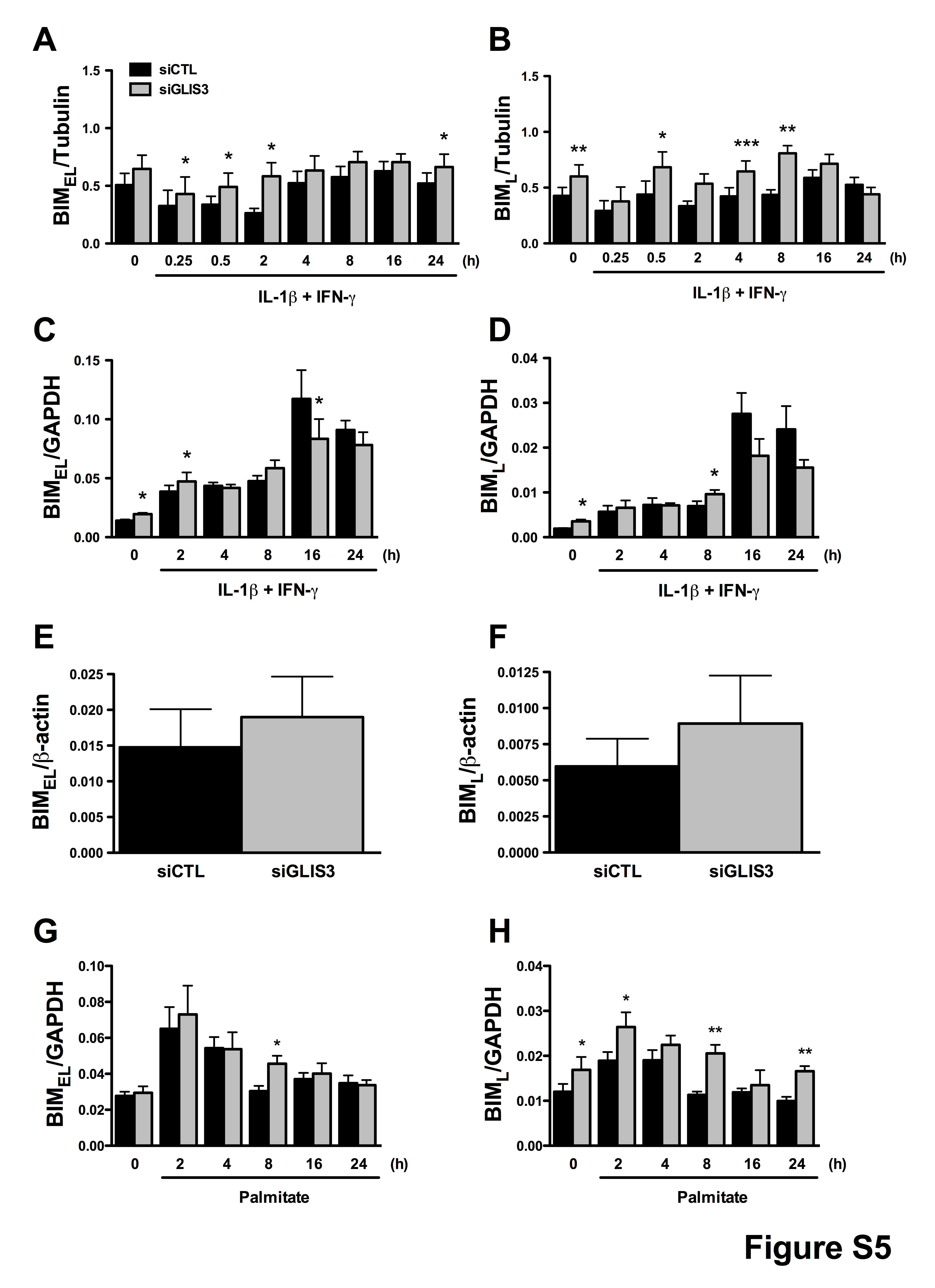

Supplement: Figure S5 — Impact of GLIS3 KD on BimEL and BimL expression in basal condition and after cytokine or palmitate treatment. After 48 h of control or GLIS3 siRNA, INS-1E cells were incubated with cytokines (A–D) or palmitate (G–H) and collected at different time points for Western blot and real-time PCR analyses. (A, B) Densitometry of BimEL and BimL protein expression normalized by α-tubulin; (C, D, G and H) mRNA expression of BimEL and BimL normalized by the housekeeping gene GAPDH. (E and F) mRNA expression in human islets of BimEL and BimL normalized by the housekeeping gene β-actin after a 48 h of control or GLIS3 siRNA transfection. Results are means ± SEM (n = 4). * P<0.05, ** P<0.01 or *** P<0.001 vs. siCTL by paired t-test. (TIF) [file pgen.1003532.s005.tif]

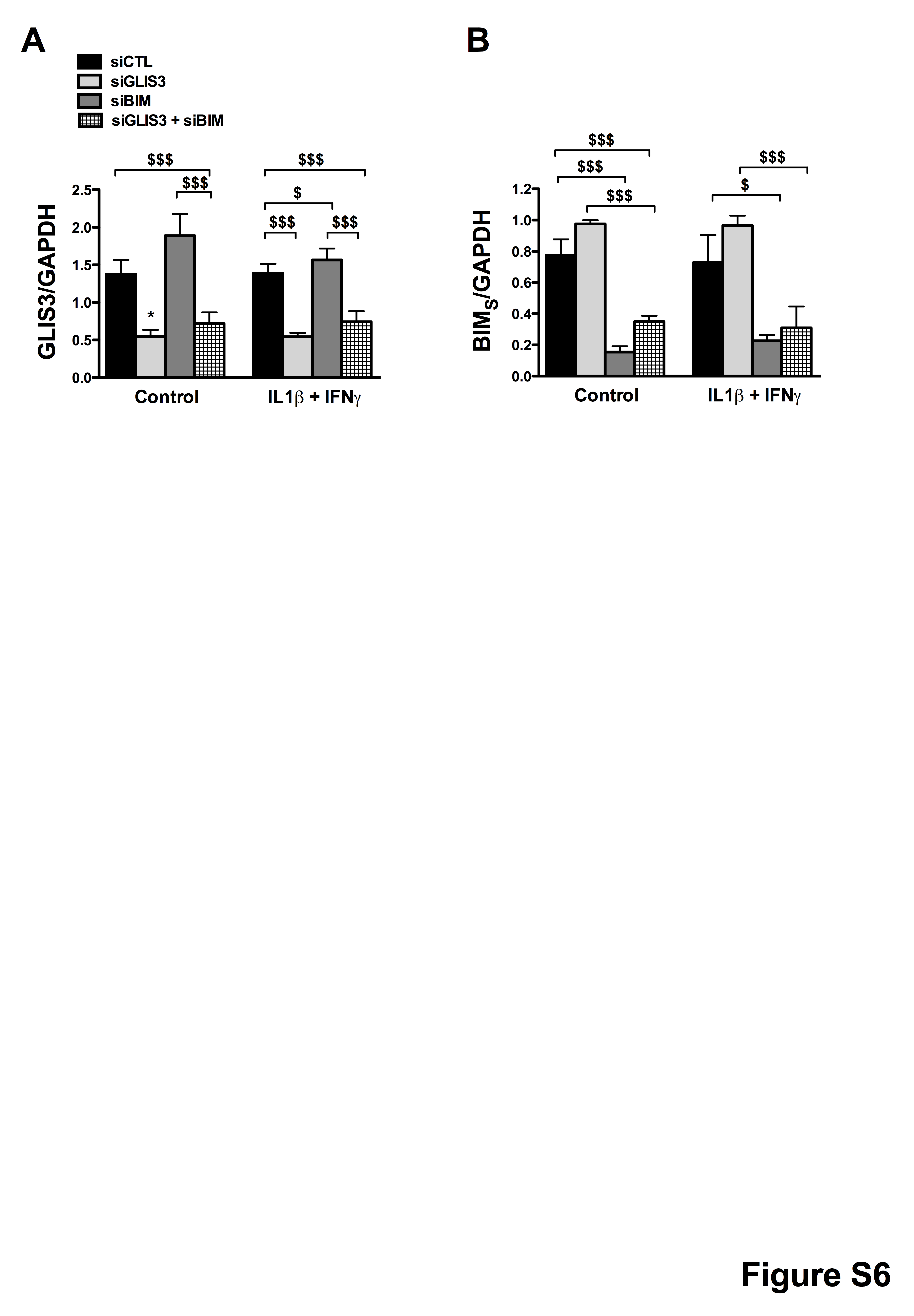

Supplement: Figure S6 — Double KD of GLIS3 and Bim in primary rat beta cells. FACS-purified rat beta cells were transfected with control, GLIS3 or Bim siRNA. After 48 h cells were treated with cytokines for 24 h. (A, B) mRNA expression of GLIS3 and BimS. Results are means ± SEM corrected by the housekeeping gene GAPDH (n = 4). * P<0.05 vs. siCTL; $ P<0.05 or $$$ P<0.001 as indicated by the bars. ANOVA followed by paired t-test with Bonferroni's correction. (TIF) [file pgen.1003532.s006.tif]

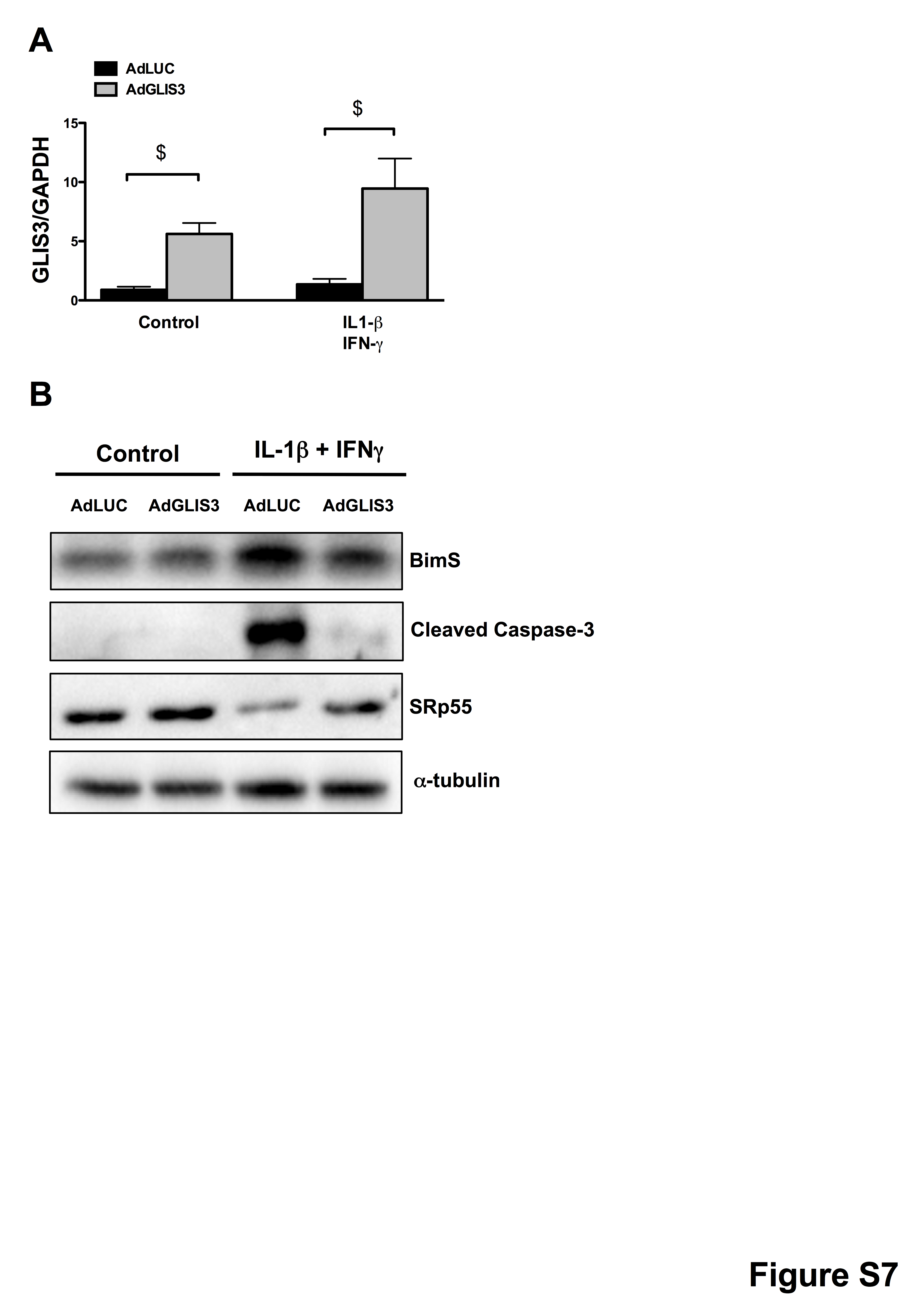

Supplement: Figure S7 — GLIS3 overexpression down regulates BimS and decreases cytokine-induced cleavage of caspase 3. INS-1E cells were infected with AdLUC or AdGLIS3 at MOI 10 and 24 h later exposed or not to cytokines for an additional 24 h. (A) mRNA expression of GLIS3 normalized by GAPDH. Results are means ± SEM (n = 4). (B) Representative blot of 2–4 independent experiments for BimS, cleaved caspase-3 and SRp55 and the housekeeping protein α-tubulin. $ P<0.05 as indicated by the bars. Paired t-test. (TIF) [file pgen.1003532.s007.tif]
