# Supplementary material for: GLIS3, a Susceptibility Gene for Type 1 and Type 2 Diabetes, Modulates Pancreatic Beta Cell Apoptosis via Regulation of a Splice Variant of the BH3-Only Protein Bim
Source: PLoS Genet. 2013 May 30;9(5):e1003532. doi: 10.1371/journal.pgen.1003532 (PMC3667755; doi:10.1371/journal.pgen.1003532)
Supplement: Table S1 — Sequences of siRNAs used to KD gene/protein expression. (DOC) [file pgen.1003532.s008.doc]

| **siRNA** | **Supplier** | **Sequence** |
| --- | --- | --- |
| **Rat** |  |  |
| Allstars Negative Control siRNA | Qiagen, Venlo, Netherlands | Not provided |
| siBim Stealth Select siRNAi Duplex Oligoribonucleotides | Invitrogen, Pasley, UK | 5´-GAGUUCAAUGAGACUUACACGAGGA-3´ |
|  |  | 5´-CGAGGAGGGCGUUUGCAAACGAUUA-3´ |
| siBim small Stealth Select siRNAi Duplex Oligoribonucleotides | Invitrogen, Pasley, UK | 5´-UGAGACUGCCUUAUGGAAGCUUGCG-3´ |
|  |  | 5´-CGCAAGCUUCCAUAAGGCAGUCUCA-3´ |
| siGLIS3 #1 Stealth Select siRNAi Duplex Oligoribonucleotides | Invitrogen, Pasley, UK | 5´-UUCACGUGCUUUCUUAGGGAGCUUG-3´ |
|  |  | 5´-CAAGCUCCCUAAGAAAGCACGUGAA-3´ |
| siGLIS3 #2 Stealth Select siRNAi Duplex Oligoribonucleotides | Invitrogen, Pasley, UK | 5´-ACAUCUUGAGGUGAAGACUGUUAGC-3´ |
|  |  | 5´-GCUAACAGUCUUCACCUCAAGAUGU-3´ |
| siSRp55 #1 Stealth Select siRNAi Duplex Oligoribonucleotides | Invitrogen, Pasley, UK | 5´-GAGCAAAUCUAAGCCCAAGUCUGAU-3´ |
|  |  | 5´- AUCAGACUUGGGCUUAGAUUUGCUC-3´ |
| siSRp55 #2 Stealth Select siRNAi Duplex Oligoribonucleotides | Invitrogen, Pasley, UK | 5´-GGAUAAGUAUGGGAAGUCACGAAGU-3´ |
|  |  | 5´-ACUUCGUGACUUCCCAUACUUAUCC-3´ |
|  |  |  |
| **Human** |  |  |
| siGLIS3 #1 Stealth Select siRNAi Duplex Oligoribonucleotides | Invitrogen, Pasley, UK | 5´-CAGCAAUAGUGUCUCUAACUCAUUA-3´ |
|  |  | 5´-UAAUGAGUUAGAGACACUAUUGCUG-3´ |
| siGLIS3 #2 Stealth Select siRNAi Duplex Oligoribonucleotides | Invitrogen, Pasley, UK | 5´-GCUGAAGAUGCUACCUUCUUGCAGA-3´ |
|  |  | 5´-UCUGCAAGAAGGUAGCAUCUUCAGC-3´ |
| siBim | Santa Cruz – sc29802 | Not provided |
