# Supplementary material for: GLIS3, a Susceptibility Gene for Type 1 and Type 2 Diabetes, Modulates Pancreatic Beta Cell Apoptosis via Regulation of a Splice Variant of the BH3-Only Protein Bim
Source: PLoS Genet. 2013 May 30;9(5):e1003532. doi: 10.1371/journal.pgen.1003532 (PMC3667755; doi:10.1371/journal.pgen.1003532)
Supplement: Table S2 — Primer sequences and their respective PCR product lengths. ST denotes standard PCR, RT denotes real time qPCR. (DOC) [file pgen.1003532.s009.doc]

| **Name** | **Forward (5´-3´)** | **Reverse (5´-3´)** | **Lenght** |
| --- | --- | --- | --- |
| **RAT** |  |  |  |
| GAPDH ST | ATGACTCTACCCACGGCAAG | TGTGAGGGAGATGCTCAGTG | 930 bp |
| GAPDH RT | AGTTCAACGGCACAGTCAAG | TACTCAGCACCAGCATCACC | 136 bp |
| Bim ST | GCCAAGCAACCTTCTGATGT | ACCAGACGGAAGATGAATCG | 572 bp |
| Bim RT | AGAGATACGGATCGCACAGG | GTCTTCCGCCTCTCGGTAAT | 100 bp |
| Bim EL RT | CCAGATCCCCACTTTTCATC | AGGACTTGGGGTTTGTGTTG | 134 bp |
| Bim L RT | CAGAATCGCAAGCTTCCATA | CTGCCTTATGGAAGCCATTG | 116 bp |
| Bim S RT | CAGAATCGCAAGCTTCCATA | GTCTTCCGCCTCTCGGTAAT | 155 bp |
| GLIS 3 ST | GGACCCATTTCACCTCCTG | ACCATGCTGTTTCCAGATCC | 755 bp |
| GLIS3 RT | AGCATGCAAGCAGATCAGTC | CCTGTAGCTCTGAGACCACT | 178 bp |
| SRP55 ST | CATAGGACGCCTGAGCTACA | ACGAACAGGTGGTCCGTATT | 307 bp |
| SRP55 RT | CATAGGACGCCTGAGCTACA | TGCCGTTCAGCTCGTAAAC | 158 bp |
|  |  |  |  |
| **HUMAN** |  |  |  |
| Bim ST | GCAAAGCAACCTTCTGATGTAA | ACACCAGGCGGACAATGTA | 310 bp |
| Bim S RT | GAGCCACAAGCTTCCATGAG | TAACCATTCGTGGGTGGTCT | 163 bp |
| Bim L RT | GACAGAGCCACAAGACAGGA | TCTTGGGCGATCCATATCTC | 162 bp |
| Bim EL RT | AGATCCCCGCTTTTCATCTT | AGGACTTGGGGTTTGTGTTG | 132 bp |
| Bim total RT | TTCTTGCAGCCACCCTGC | CTTGCGTTTCTCAGTCCGA | 152 bp |
| GLIS3 ST | CCAGCAAGCCAGGTCTCTAC | GGATGAGTGCCGAGGACTAG | 372 bp |
| GLIS3 RT | CAACCAGATCAGTCCTAGCTTACA | GCGAAATAAGGGACCTGGTATC | 86 bp |
| ACTB ST | AAATCTGGCACCACACCTTC | CCGATCCACACGGAGTACTT | 805 bp |
| ACTB RT | CTGTACGCCAACACAGTGCT | GCTCAGGAGGAGCAATGATC | 127 bp |
